# Electrically silent mutants unravel the mechanism of binding−gating coupling in Cys-loop receptors

**DOI:** 10.1126/sciadv.adq8048

**Published:** 2024-11-27

**Authors:** Nicole E. Godellas, Gisela D. Cymes, Claudio Grosman

**Affiliations:** ^1^ Department of Molecular and Integrative Physiology, University of Illinois at Urbana-Champaign, Urbana, IL 61801, USA.; ^2^ Center for Biophysics and Quantitative Biology, University of Illinois at Urbana-Champaign, Urbana, IL 61801, USA.; ^3^ Neuroscience Program, University of Illinois at Urbana-Champaign, Urbana, IL 61801, USA.

## Abstract

The transduction of extracellular chemical signals into intracellular events relies on the communication between neighboring domains of membrane receptors. In the particular case of Cys-loop receptor channels, five short stretches of amino acids, one per subunit, link the extracellular and transmembrane domains in such a way that the ion permeability of the latter and the affinity for neurotransmitters of the former become tied to each other. Here, using direct functional approaches, we set out to understand the molecular bases of this crucial interdependence through the characterization of total loss-of-current mutations at the interface between domains. Our results indicate that domain−domain proximity plays a previously unnoticed critical role inasmuch as inserting a single residue in each linker rendered the two domains independent of each other. In marked contrast, loss-of-current mutations that leave the linkers’ length unaltered did not compromise the interdomain coupling, but rather, seemed to cause agonist-bound closed receptors to desensitize without appreciably opening.

## Teaser

Signal transduction through Cys-loop receptors hinges on the proximity between the extracellular and transmembrane domains.

## INTRODUCTION

Neurotransmitter-gated ion channels (NGICs) transduce the binding of extracellular ligands to the opening of a transmembrane pore. NGICs achieve this by being able to adopt distinct, interconvertible conformations that differ not only in the permeability of the pore to ions but also in the affinity of the binding sites for neurotransmitter (*1*). These conformations (“states”) are known as “closed” (low affinity, nonconductive), “open” (high affinity, ion conductive), and “desensitized” (high affinity, nonconductive). Furthermore, these receptor channels can be bound to neurotransmitter or unbound (“unliganded”). When unliganded, the closed conformation is, by far, the most stable, but because the affinity for neurotransmitter is much higher in the open and desensitized states, the conformational free-energy landscape changes upon binding neurotransmitter. When fully liganded and at equilibrium, it is the desensitized conformation that dominates, instead; the open conformation is only transiently visited as the channel switches from the closed to the desensitized state. Central to this mechanism is the idea that binding site affinities and the ion-conductive/nonconductive states of the pore are tied to each other. Certainly, an NGIC whose neurotransmitter-binding sites are in a low-affinity conformation has a closed-type nonconductive pore, whereas an NGIC whose neurotransmitter-binding sites are in a high-affinity conformation has an ion-conductive, open pore or a desensitized-type, nonconductive pore. Although brief-lived conformations with hybrid properties are likely to be visited as the protein traverses the rugged free-energy landscape connecting these end states, our work here is concerned only with the physiologically well-defined closed, open, and desensitized states.

**Fig. 1.**
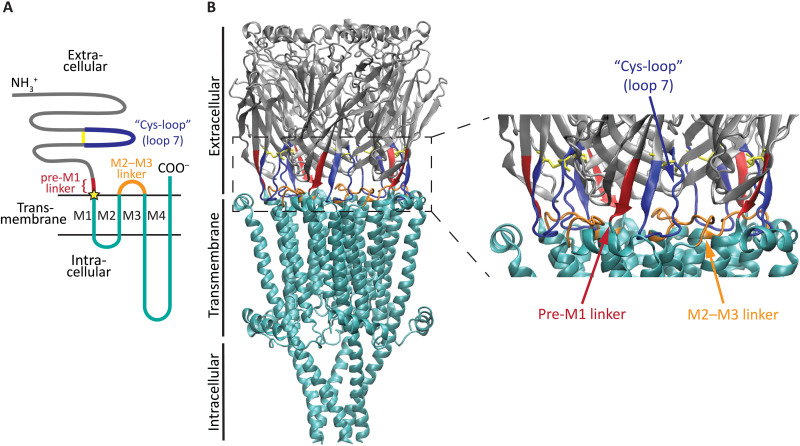
The ECD−TMD interface of pLGICs. (**A**) Membrane-threading pattern common to all pLGIC subunits. The Cys loop, the pre-M1 linker, and the M2−M3 linker, that is, the three stretches of ECD residues mutated in this work, are schematically indicated. A star symbol indicates the ECD–TMD junction site. (**B**) The Cys loop, the pre-M1 linker, and the M2−M3 linker mapped onto an atomic model of the unliganded human α7-AChR [PDB ID code 7EKI (*72*)]. Molecular images were made with Visual Molecular Dynamics [VMD (*73*)] using ribbon representation.

All NGICs share, essentially, the same modular design consisting of an extracellular domain (ECD) that harbors the neurotransmitter-binding sites and a transmembrane domain (TMD) that forms the ion-permeable pore. This modular architecture is particularly evident in the case of the superfamily of pentameric ligand-gated ion channels (pLGICs; also known as Cys-loop receptors) where the ECD and TMD are formed by the N- and C-terminal “halves” of each subunit, respectively (Fig. 1A) (*2*). Crucially, the five short stretches of amino acids that bring the two modules together [the “pre-M1 linkers” (*3*); Fig. 1B] do so in such a way that the conformations of the linked domains, and hence, their functional properties, depend tightly on each other’s. Intriguingly, chimeric combinations of wild-type ECDs and TMDs from distantly related members of the superfamily often result in receptor channels that display all the functional properties characteristic of naturally occurring pLGICs [e.g., (*4*–*11*)]. Other elegant examples of the structural and functional modularity of pLGICs are: (i) the occurrence, in some invertebrates, of ECD-only pentameric acetylcholine- (ACh) binding proteins (*12*); and (ii) the ability of isolated pore-lining M2 α-helices (Fig. 1A) to self-assemble in membranes and form charge-selective pores that can open and close (*13*, *14*). The question then arises as to how the seemingly independent ECD and TMD modules behave as a unit once covalently linked.

Several different mechanisms have been proposed to underlie the “coupling” between the affinity of the ECD’s neurotransmitter-binding sites and the ion permeability of the TMD’s pore, but details aside, they all converge into the notion that interactions between wild-type side chains across the ECD−TMD interface are key to this phenomenon [e.g., (*3*, *4*, *7*, *9*, *11*, *15*–*20*)]. In this context, the successful design of fully functional ECD−TMD chimeras has frequently been attributed to the residual conservation of sequence that often exists at the level of the domain−domain interface, even between evolutionarily distant parent channels. We have recently challenged this idea with the generation of fully functional chimeric pLGICs [having the ECD of the α7 nicotinic ACh receptor (α7-AChR) from chicken (*21*) and the TMD of the β-subunit of *Caenorhabditis elegans*’ glutamate-gated Cl^−^ channel (*22*) (β-GluCl)] mutated so as to remove traces of sequence conservation across the ECD−TMD interface (*23*). The complete elimination of this chemical complementarity upon mutation of all conserved residues at one side of the interface leads to a construct that retains all the functional properties expected from naturally occurring members of the superfamily; we refer to this construct as “NWCL” for “no wild-type contact left” (fig. S1). Hence, the conservation of physicochemical properties across the domain−domain interface, let alone, the conservation of specific amino acid residues, is not required for binding−gating coupling. However, if specific side chain–side chain interactions across the interface are not required for the communication between domains, then, what is?

Although ion channel gating proved to be highly resilient to mutations to the ECD−TMD interface of the α7-AChR–β-GluCl chimera, we did identify mutations in this region of the protein that rendered the channel electrically silent (*23*). That is, currents could not be recorded from these mutants even though they expressed well on the plasma membrane. Here, we aimed to learn about the molecular basis of binding−gating signal transduction in pLGICs through the understanding of this phenotype. Do these mutations sever the coupling between the binding sites and the pore in such a way that the ECD and the TMD lose their conformational interdependence? Are these mutations, instead, extreme loss-of-function perturbations that stabilize (“lock”) the receptor in the low-affinity–closed-channel conformation to the extent that agonists can no longer open it? Or do these mutants desensitize directly from the closed state, skipping sojourns in the open-channel conformation altogether? Even more elementarily: Do these mutants bind orthosteric ligands at all?

To address these questions, we applied a recently developed ligand-binding assay approach that allows the study of pLGIC function when, as was the case here, measuring ion transport is not an option (*24*). We probed the interdependence of the ECD and TMD by estimating the effect of molecular perturbations to the latter on the ligand-binding properties of the former. We found that single-residue insertions in the pre-M1 linker, at the ECD−TMD junction site (Fig. 1), cause well-coupled receptor channels to become two independent modules. Conversely, we found that loss-of-current mutations that leave the length of the pre-M1 linkers unaltered do not compromise the interdependence of the two domains, but rather, seem to cause agonist-bound closed channels to desensitize without appreciably opening.

In light of these results, we propose that a specific, narrowly defined number of amino acid residues linking any two wild-type pLGIC ECD and TMD modules may well be all that is required to ensure their conformational coupling. It follows that bidirectional ECD−TMD communication in pLGICs relies more heavily on domain−domain proximity than it does on chemical complementarity or electrostatic matching across the interface.

## RESULTS

In a previous study of the chimera containing the ECD of the α7-AChR from chicken and the TMD of β-GluCl from *C. elegans*, we identified three mutations at the ECD−TMD interface that render the channel electrically silent (*23*). These are (Fig. 1): (i) an eight-residue mutation that replaces the chimera’s β-GluCl’s M2−M3 linker (LPPVSYVK) with that of the α7-AChR’s (MPATSDSV), thus generating a channel whose ECD and TMD interfacial loops (that is, loop 2, Cys loop, and loop 9 from the ECD, and the M2−M3 linker from the TMD) belong to the same channel; (ii) mutation of the phenylalanine (to alanine; F157A) that precedes the universally conserved proline of the Cys loop (*25*); and (iii) the insertion of a fourth arginine in the α7-AChR’s RRR motif that immediately precedes the chimeric junction site (the pre-M1 linker). Because these mutations map to the domain−domain interface, we reasoned that elucidating the molecular basis of their loss-of-current effect could illuminate the mechanism of binding−gating coupling in pLGICs. Since probing function using electrophysiology was not an option, we resorted to equilibrium ligand-binding competition assays (*24*).

In our experiments, the chimeric receptor was present on the plasma membrane of transfected human embryonic kidney (HEK)–293 cells, and the labeled ligand was radioiodinated α-bungarotoxin {[^125^I]-α-BgTx (*26*)} at a concentration that (in the absence of competing unlabeled ligand) half-saturates the receptor. α-BgTx is a (membrane-impermeant) 74-amino-acid snake neurotoxin that binds to the neurotransmitter-binding sites of muscle and α7-AChRs in a manner that is mutually exclusive with the binding of orthosteric ligands (*21*, *27*, *28*). Moreover, α-BgTx acts as an inverse agonist, that is, it binds with higher affinity to (and thus favors) the resting, closed-channel conformation of the receptor (*29*–*31*). The α7-AChR–β-GluCl chimera displays all the ligand-binding (*24*) and ion-channel gating (*23*) properties that are expected from wild-type members of the superfamily.

In what follows, we interpreted the results of our ligand-binding assays in terms of the elementary steps of thermodynamic cycles of ligand-gated ion-channel activity (fig. S2), namely, gating conformational changes and individual ligand-binding steps (*1*). Furthermore, on the basis of previous experimental results with this chimeric construct (*24*), all five orthosteric ligand–binding sites were considered to be identical and independent of each other’s occupancy. In this framework, the gating equilibrium constant of the channel in any of its different ligand-bound states (that is, monoliganded, diliganded, triliganded, and so on; *K*_C*m*⇌O*m*_) can be thought of as being determined by the three “independent” variables of the system, namely, the gating equilibrium constant of the unliganded receptor (*K*_C⇌O_), the ligand-dissociation equilibrium constant from the closed-channel conformation (*K*_D,closed_), and the ligand-dissociation equilibrium constant from the open-channel conformation (*K*_D,open_)

$$K_{C_{m}⇌O_{m}}=K_{C⇌O}\times {\left(\frac{K_{D,\text{closed}}}{K_{D,\text{open}}}\right)}^{m}$$ (1)

where *m*, an integer from 1 to 5, is the number of ligand molecules bound. For the sake of simplicity, the open and desensitized conformations, that is, the conformations of the channel that bind agonists with higher affinity, were grouped together and are collectively referred to as “open.” To our knowledge, whether the affinities of the open and desensitized conformations of the α7-AChR (or any other pLGIC) for agonists are identical or different remains unsettled; we only know that, for any agonist, both must be higher than that of the closed-channel conformation.

### Probing function in electrically silent pLGICs

**Fig. 2.**
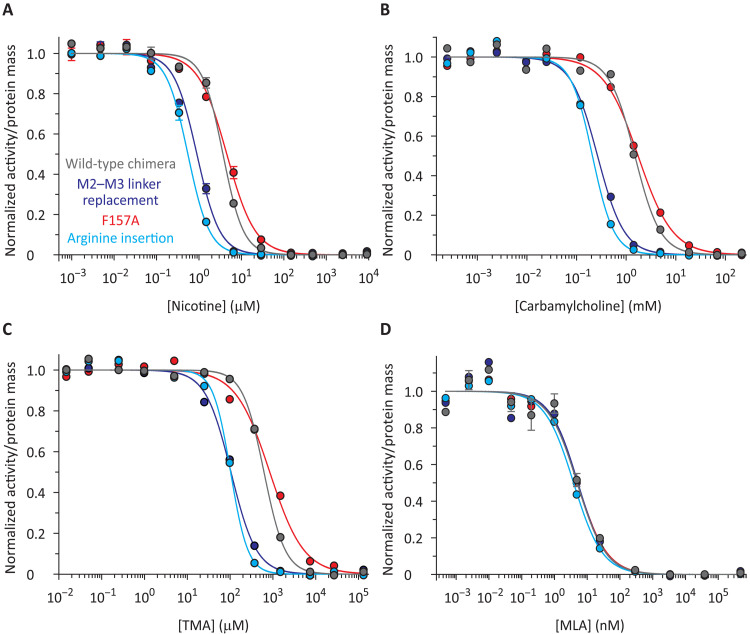
Binding competition curves of the “wild-type” α7-AChR–β-GluCl chimera and three electrically silent mutants. (**A**) Nicotine. (**B**) Carbamylcholine. (**C**) TMA. (**D**) MLA. The curves were fitted with single-component Hill equations (table S1), and for all of them, the labeled ligand was [^125^I]-α-BgTx at a concentration (of the unbound form) of ~1 × *K*_D,closed_ (fig. S3). The color code is the same for all panels.

Figure 2 shows that the three mutants bind orthosteric ligands well (see also fig. S3). Certainly, as their concentrations rose, the α7-AChR agonists nicotine, carbamylcholine, and tetramethylammonium (TMA), as well as the inverse agonist methyllycaconitine [MLA (*29*)], outcompeted the binding of α-BgTx. Moreover, as expected from pLGICs that predominantly occupy the low-affinity–closed-channel (“resting”) conformation when unliganded and that have identical and independent orthosteric binding sites: (i) the corresponding concentration−response curves were best-fitted with single-component Hill equations; and (ii) the estimated values of the Hill coefficients were >1 for the agonists, and ~1 for the inverse agonist (table S1). These findings are a strong indication that, under the conditions of our assays (24- or 48-hour incubations at 37°C), equilibrium was closely approached between the receptor and the competing ligands (*24*).

The effect of each mutation on the concentration−response curves was largely the same irrespective of the particular agonist used to compete against α-BgTx (table S1). For the arginine insertion mutant, the half-competition concentration of unlabeled ligand decreased (relative to that of the “wild-type” chimera) by a factor of 6.7 ± 0.36 for nicotine, 7.2 ± 0.64 for carbamylcholine, and 6.0 ± 0.48 for TMA (uncertainties calculated by error propagation). Similarly, for the M2−M3 linker replacement mutant, the half-competition concentration of unlabeled ligand decreased by a factor of 4.1 ± 0.29 for nicotine, 5.7 ± 0.47 for carbamylcholine, and 5.9 ± 0.47 for TMA. For the F157A mutant, on the other hand, the half-competition concentration increased for the three agonists (by a factor of only 1.2 ± 0.08 for nicotine, 1.2 ± 0.09 for carbamylcholine, and 1.4 ± 0.14 for TMA). As for the Hill coefficient, the observed higher-than-unity values obtained for all nine combinations of mutants and agonists (table S1) suggest that none of the mutations prevented the orthosteric ligand-binding sites from undergoing the low- to high-affinity interconversion as they transitioned from being half-bound to α-BgTx (at one end of the curve) to being fully bound to unlabeled agonist (at the other end of the curve). In other words, none of the studied mutations seem to have locked the receptor in the low-affinity–closed-channel state or the (equally nonconductive) high-affinity–desensitized state. As recently discussed (*24*), the Hill coefficient (*n*_H_) of a binding competition assay can be regarded as an indicator of the extent to which a receptor channel changes its affinity for the unlabeled ligand in going from one end of the competition curve to the other. Thus, *n*_H_ ≈ 1 indicates that the affinity remains the same throughout the curve. On the other hand, 1 < *n*_H_ < *n* (where *n* is the total number of binding sites) indicates that the receptor undergoes conformational changes that alter the affinity for the unlabeled ligand as the latter binds to the receptor, outcompetes the labeled toxin, and eventually occupies all the available sites.

For MLA, the half-competition concentration values and the Hill coefficients of all three mutants remained close to those of the wild type (~5 nM and ~1.0, respectively; table S1). Because the half-competition concentration of an inverse agonist is a direct probe of its dissociation equilibrium constant from the closed-channel conformation (under the particular conditions of our experiments, [Half-competition] = 2 × *K*_D,closed_; [fig. S2; (*24*)], these results indicate that none of the mutations changed the affinity of the binding sites for MLA. Therefore, it would be intriguing to extend this conclusion to the three agonists tested here and suggest that the mutations did not have large effects on their binding site affinities, either. If this were the case, then, the displacement of the mutant competition curves along the concentration axis (Fig. 2) would be the result of changes only in the low-affinity ⇌ high-affinity (“gating”) equilibrium constant of the unliganded receptor.

### Probing domain−domain coupling with TMD-binding allosteric modulators

**Fig. 3.**
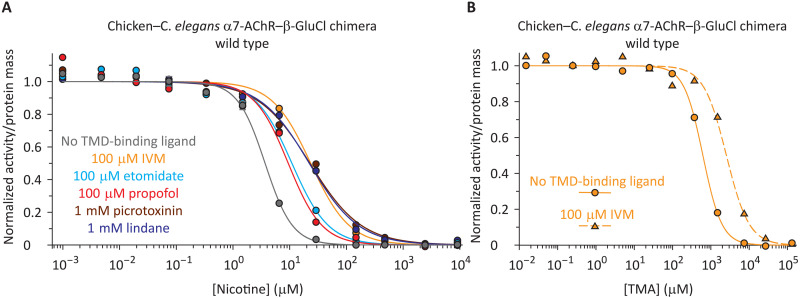
Binding competition curves of the α7-AChR–β-GluCl chimera are sensitive to TMD-binding ligands. (**A**) Nicotine. (**B**) TMA. All five allosteric modulators shifted the curves to higher concentrations of nicotine or TMA, an observation that characterizes them as negative allosteric modulators. Note that IVM is a positive allosteric modulator, instead, of the closely related α-GluCl homomer and the α–β-GluCl heteromer (*22*). The curves were fitted with single-component Hill equations (table S1), and for all of them, the labeled ligand was [^125^I]-α-BgTx at a concentration (of the unbound form) of ~1 × *K*_D,closed_ (fig. S3). The structures of the TMD-binding ligands are shown in fig. S4.

Although the results above indicate that the orthosteric sites are fully functional in the three electrically silent mutants, they tell us nothing regarding whether they remain coupled to the pore domain or not. To this end, we sought to probe the conformational interdependence between the ECD and the TMD by assessing the effect of TMD-binding allosteric ligands on the competition between labeled and unlabeled (ECD-binding) orthosteric ligands. Not much is known about ligands that bind to the TMD of *C. elegans* β-GluCl other than the notion (derived from electrophysiological observations) that ivermectin (IVM) and barbiturates act as negative allosteric modulators (*32*–*34*), and that picrotoxinin is as an open-channel pore blocker (*35*). Figure 3A shows the competition curves between nicotine and α-BgTx for the (wild-type) α7-AChR–β-GluCl chimera in the presence of 100 μM IVM, 100 μM etomidate, 100 μM propofol, 1 mM picrotoxinin, or 1 mM lindane (figs. S4 and S5). IVM (*36*), etomidate (*37*, *38*), and propofol (*37*) are expected to bind to the outer lining of β-GluCl’s TMD pore, whereas picrotoxinin (*35*, *39*) and lindane (*40*) are expected to bind inside the pore. All five compounds shifted the curves to higher concentrations of nicotine (table S1). To extend these observations to other small-molecule orthosteric ligands, we also tested the effect of 100 μM IVM on the competition between α-BgTx and TMA on the wild-type chimera; we found IVM’s effect to be largely the same whether nicotine or TMA was the competing orthosteric ligand (Fig. 3B and table S1).

**Fig. 4.**
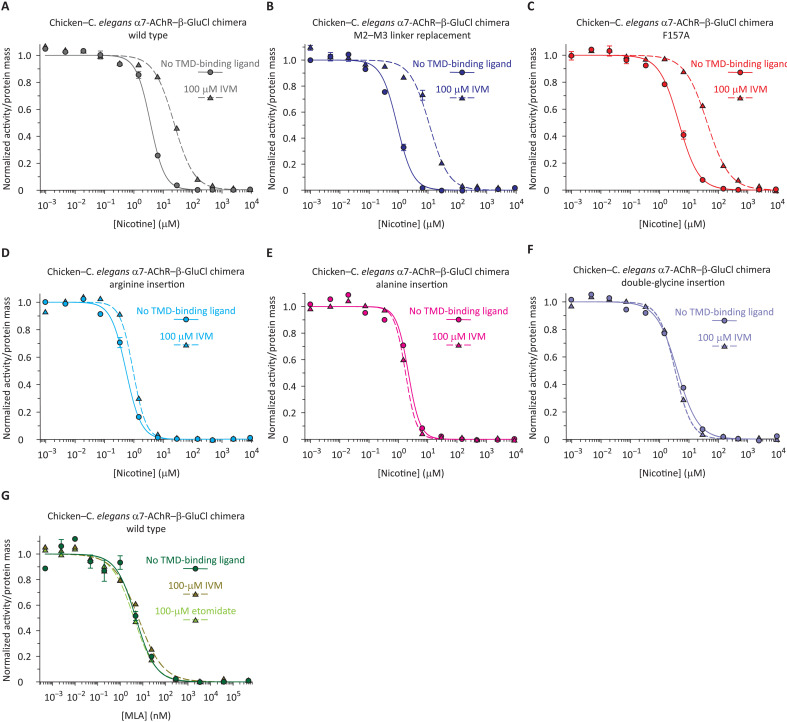
Probing ECD–TMD coupling in the α7-AChR–β-GluCl chimera using IVM. (**A** to **F**) α-BgTx–nicotine curves of the indicated constructs. The insertion of one or two residues in the pre-M1 linker markedly reduced the rightward shift caused by IVM. (**G**) α-BgTx–MLA curves of the wild-type construct. The curves were fitted with single-component Hill equations (table S1), and for all of them, the labeled ligand was [^125^I]-α-BgTx at a concentration (of the unbound form) of ~1 × *K*_D,closed_ (fig. S3).

The right-shifting effect of the tested β-GluCl TMD-binding compounds on the competition curves was taken as an indication of ECD−TMD interdependence. Because of its large effect, we chose to use IVM to probe the domain−domain coupling of the three electrically silent mutants. In the presence of 100 μM IVM, the half-competition concentration of nicotine increased by a factor of 6.4 ± 0.55 for the wild-type chimera, 13 ± 1.2 for the M2−M3 linker mutant, and 9.2 ± 0.82 for the F157A mutant (Fig. 4, A to C, and table S1). These findings indicate that, despite not conducting currents, the ECD and TMD of these two mutants remain coupled. In other words, loss-of-current mutations at the ECD−TMD interface do not necessarily disrupt the communication between the two domains. Instead, we propose that these mutations may cause agonist-bound closed channels to desensitize without appreciably opening. This seems to be the most plausible mechanism underlying the effect of mutations that render ligand-gated ion channels electrically silent without preventing the ECD’s low-affinity ⇌ high-affinity interconversion and without severing the coupling between domains. In marked contrast, however, the half-competition concentration of nicotine for the arginine insertion mutant increased by a factor of only 1.7 ± 0.12 (Fig. 4D and table S1), which suggests that this insertion greatly weakened, or even eliminated, the interdependence between the ECD and the TMD.

To learn whether inserting residues other than arginine between the two domains also causes the ECD−TMD coupling to be lost, we inserted alanines and glycines. Whereas insertions right before the first arginine of the pre-M1 linker led to a drastic decrease in plasma membrane expression, insertions after the third arginine were better tolerated (table S2), and their effect on function could be studied in detail. From both electrophysiological and ligand-binding assay perspectives, the single-alanine and the double-glycine insertion mutants (RRR**A** and RRR**GG**) behaved like the mutant having a single-arginine insertion (RRRR). Certainly, ACh concentration jumps from 0 to 100 μM failed to elicit currents from these two mutants (*n* = 10 different whole-cell patches for the alanine insertion mutant, and *n* = 18 for the double-glycine insertion mutant) even though they expressed well on the plasma membrane. Moreover, the corresponding α-BgTx–nicotine competition curves were best fitted with single-component Hill equations having larger-than-unity Hill coefficients and were essentially insensitive to the presence of 100 μM IVM (Fig. 4, E and F). The half-competition concentration of nicotine decreased by a factor of only 1.3 ± 0.10 for the single-alanine insertion and 1.2 ± 0.11 for the double-glycine insertion (table S1). These results, along with the previous observation that residue-to-residue mutations of the first and third arginines of the RRR pre-M1 linker were functionally well-tolerated (*23*), lead us to propose that it is the increased length of the interdomain linker (rather than its mere mutation) that causes the uncoupling of the two domains. Furthermore, inasmuch as the double-glycine insertion had the same effect as the single-arginine and single-alanine insertions, it seems as though inserting a single residue per subunit suffices to “disconnect” the two domains. Lengthening the pre-M1 linker is likely to increase the physical separation between the ECD and the TMD, as well as alter the spatial orientation and dynamics of the two domains relative to each other. As for the functional impact of shortening the pre-M1 linker, low levels of expression precluded a detailed mechanistic analysis. Deleting one of the three arginines lowered plasma membrane expression by a factor of ~90 (and currents could not be recorded; *n* = 10), whereas deleting single residues preceding the R^227^RR motif (Thr^225^ or Met^226^) lowered plasma membrane expression to undetectable levels (table S2).

We conclude that, as is the case for the wild-type chimera, the ECDs of the insertion mutants transition from the low-affinity to the high-affinity conformation as the concentration of nicotine increases from negligible to saturating. However, unlike the wild-type constructs, the TMDs of the insertion mutants do not accompany this change of the ECD. The bidirectional ECD ⇌ TMD communication is lost; the two domains become effectively uncoupled.

### A digression on the mechanisms of action of TMD-binding allosteric modulators

In the presence of the tested TMD-binding allosteric ligands, the wild-type-chimera’s competition curves between α-BgTx and unlabeled orthosteric ligands shifted to the right (Figs. 3 and 4A). As is the case for any perturbation to the system, this displacement of the curves to higher concentrations could be the result of: (i) a decreased low-affinity ⇌ high-affinity gating equilibrium constant of the orthosteric ligand-free receptor; and/or (ii) changes in the affinities for the orthosteric ligand (*24*). To distinguish between these two possibilities, we resorted to the use of MLA. We performed α-BgTx–MLA competition assays using the wild-type chimera in the presence of IVM or etomidate (Fig. 4G). As previously mentioned, because MLA is an inverse agonist (*29*) and the wild-type chimera is predominantly closed when unliganded, these curves specifically probe the binding of MLA to the closed state of the receptor. That is, when MLA is used as the competing orthosteric ligand, affinity-switching conformational changes are not expected to occur in going from one end of the competition curve to the other. As illustrated in Fig. 4G, we found that IVM (100 μM) and etomidate (100 μM) have little, if any, effect on the half-competition concentration of MLA (table S1), thus suggesting that an effect of these TMD-binding ligands on the receptor’s affinities for orthosteric ligands is unlikely. Instead, these observations support the notion that IVM and etomidate affect the equilibrium of the low-affinity ⇌ high-affinity interconversion of the (orthosteric ligand-free) chimera. It is likely that all ligands binding to the TMD of pLGICs act in this manner, namely, by stabilizing/destabilizing the closed, open, and desensitized conformations of the channel relative to each other without affecting orthosteric site ligand-binding affinities. These results add mechanistic insight to the electrophysiological description of IVM as a negative allosteric modulator of β-GluCl (*33*, *34*). Such an inhibitory effect could have arisen from a number of molecular effects, and our results narrowed these possibilities down to only one: IVM favors the closed state of β-GluCl. Also, we notice that the effect of picrotoxinin on the conformational equilibria of the chimera resembles the closed-state stabilizing effect of picrotoxinin block on γ-aminobutyric acid receptors (GABA_A_Rs) (*41*, *42*).

Last, to better illustrate the interaction between orthosteric and allosteric ligands, we performed chemical-equilibrium calculations in the framework of a kinetic model with five orthosteric sites, five allosteric sites, and two receptor conformations. Figure S6 shows the expected effect of state-stabilizing allosteric ligands on the competition between (labeled and unlabeled) orthosteric ligands. Different allosteric ligand concentrations and different extents of state-specific stabilization were used in the calculations. The figure also quantifies the binding and unbinding of allosteric ligand (present at a constant concentration) as the receptor channel transitions from one end of the binding competition curve to the other.

### Probing channel opening with an open-channel pore blocker

Because domain−domain coupling was conserved in the two mutants that retained the wild-type length of the pre-M1 linker (Fig. 4, A to C, and table S1), we hypothesized that their electrically silent phenotype may well result from the “low open-state occupancy” mechanism. To challenge the idea of an open state that is only fleetingly occupied, infrequently visited, or that is circumvented altogether as agonist-bound closed channels desensitize, we resorted to picrotoxinin.

Electrophysiological evidence indicates that picrotoxinin binds to and unbinds from the transmembrane pore of β-GluCl homomers only in the open-channel conformation (*35*). Moreover, we found that picrotoxinin blockade of the α7-AChR–β-GluCl chimera shifts the α-BgTx–nicotine competition curve to higher concentrations (Fig. 3A and table S1), and we concluded that this shift reflects the closed-state stabilizing effect of the blocker. Therefore, since bound picrotoxinin favors channel closure and channel closure prevents picrotoxinin’s escape from the pore, picrotoxinin promotes its own trapping. This means that, after the long, 24-hour incubations with a millimolar concentration of picrotoxinin, the latter could be bound to a considerable fraction of the channels even if their open probability were very low.

In the presence of a saturating concentration of picrotoxinin [1 mM (*35*)], the half-competition concentration of nicotine increased by a factor of 6.2 ± 0.73 for the wild-type chimera (Fig. 3A and table S1), 3.1 ± 0.3 for the F157A mutant, and 1.8 ± 0.26 for the M2−M3 linker replacement mutant (fig. S7, A to C, and table S1). The finding that the competition curves of the two mutants shifted to higher concentrations indicates that they can open, at least, to some extent. On the other hand, the finding that the right-shifting effect of picrotoxinin on the mutants’ curves is more limited than it is on the wild-type’s is consistent with these two mutants occupying the open state with much lower probability in going from one end of the competition curve to the other. In turn, this is entirely consistent with the failure of ACh-concentration jumps to elicit robust currents from these mutants in patch-clamp experiments (*23*).

As negative controls, we also tested the effect of 1 mM picrotoxinin on the arginine and the alanine insertion mutants. Inasmuch as the ECD and TMD of these two constructs are uncoupled, their respective binding competition curves were expected to be insensitive to the presence of picrotoxinin. Gratifyingly, 1 mM picrotoxinin had, essentially, no effect; the half-competition concentration of nicotine increased by a factor of only 1.1 ± 0.09 for the arginine insertion mutant and decreased by a factor of only 1.6 ± 0.14 for the alanine insertion mutant (fig. S7, D and E, and table S1).

### Extending our observations to the wild-type α7-AChR

**Fig. 5.**
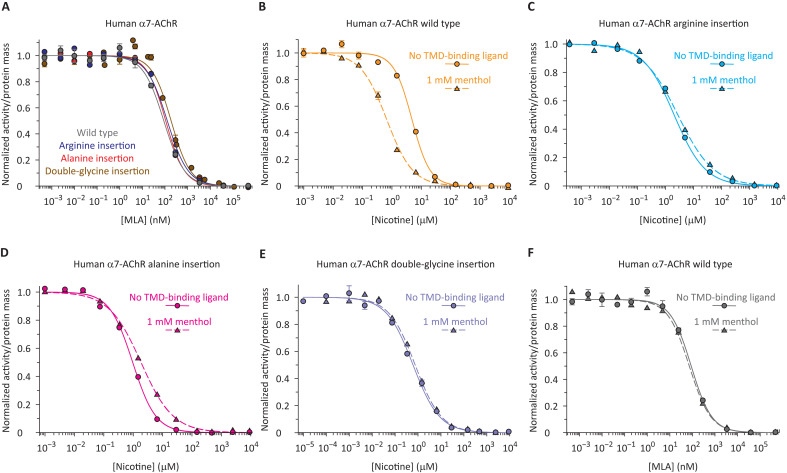
Probing ECD–TMD coupling in the human α7-AChR using menthol. (**A**) Lack of effect of residue insertions in the pre-M1 linker on the affinity of the channel’s closed state for MLA. (**B** to **E**) Effect of menthol on the α-BgTx–nicotine competition curves. The insertion of residues in the pre-M1 linker reduced the shift caused by menthol. (**F**) Lack of effect of menthol on the affinity of the wild-type’s closed state for MLA. The curves were fitted with single-component Hill equations (table S1), and for all of them, the labeled ligand was [^125^I]-α-BgTx at a concentration (of the unbound form) of ~1 × *K*_D,closed_ (fig. S3). Unlike the competition curves obtained with the α7-AChR–β-GluCl chimera, some of the curves obtained with the full-length α7-AChR required Hill coefficients that were lower than unity to be best-fitted. Although different phenomena can underlie this finding (*24*), we tentatively favor the idea that this construct may approach equilibrium more slowly.

It could be argued that the low sequence complementarity across the domain−domain interface of an ECD−TMD pLGIC chimera results in weakened trans-domain interactions that may predispose the channel to uncoupling. To test this idea, we lengthened the pre-M1 linker of the homomeric, full-length human α7-AChR, as well. We inserted one (an arginine or an alanine) or two residues (glycines) after the third arginine of the pre-M1 linker, much like we did with the chimera. We found that, despite expressing well on the plasma membrane (table S2), robust currents could not be recorded from these insertion mutants (*n* = 33 for the arginine insertion, *n* = 20 for the alanine insertion, and *n* = 20 for the double-glycine insertion), even in the presence of a saturating concentration (3 μM) of extracellular PNU-120596 [(*43*); fig. S4], a TMD-binding “positive allosteric modulator” that slows down entry into desensitization (*n* = 23 for the alanine insertion mutant, the best-expressing insertion mutant tested here; table S2). We also found that MLA (Fig. 5A) and nicotine (Fig. 5, B to E) fully outcompete the binding of α-BgTx to the three insertion mutants, and that the latter’s closed-state affinities for MLA are, essentially, wild-type like (table S1).

To probe the conformational interdependence between the ECD and the TMD, we used the α7-AChR TMD-binding ligand menthol (*44*). We favored the use of menthol (1 mM) over the use of PNU-120596 [a much more widely used allosteric modulator in the context of electrophysiological recordings (*43*)] because of its larger left-shifting effect on the ligand-binding competition curves (fig. S8 and table S1). Whereas, for the wild-type α7-AChR, 1 mM menthol decreased the half-competition concentration of nicotine by a factor of 7.3 ± 0.58, 1 mM menthol shifted this concentration by a factor of only 1.4 ± 0.23 for the arginine insertion mutant, 1.9 ± 0.23 for the alanine insertion mutant, and 1.2 ± 0.19 for the double-glycine insertion mutant (Fig. 5, B to E). Furthermore, as was the case for IVM on the α7-AChR–β-GluCl chimera, menthol had, essentially, no effect on the closed-state affinity of the human α7-AChR for MLA (Fig. 5F and table S1). Collectively, these results indicate that increasing the length of the five pre-M1 linkers by one residue suffices to uncouple the ECD and TMD of both chimeric and naturally occurring pLGICs. Moreover, they suggest that menthol biases the conformational equilibria of the α7-AChR without affecting state-specific affinities for molecules that bind to the orthosteric sites. Much more generally, these results lend ample credence to the use of ECD−TMD chimeric constructs, especially when it comes to elucidating the mechanistic aspects of ECD−TMD communication in this superfamily of ion channels.

**Fig. 6.**
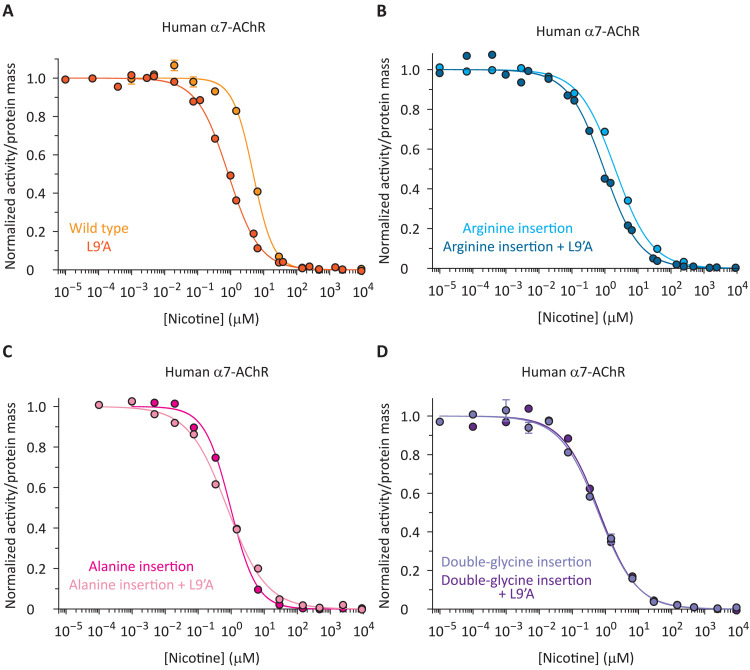
Probing ECD–TMD coupling in the human α7-AChR using the gain-of-function mutation L9′A. (**A** to **D**) Effect of the L9′A mutation on the α-BgTx–nicotine competition curves. The insertion of residues in the pre-M1 linker reduced the shift caused by the mutation. The curves were fitted with single-component Hill equations (table S1), and for all of them, the labeled ligand was [^125^I]-α-BgTx at a concentration (of the unbound form) of ~1 × *K*_D,closed_ (fig. S3).

It could also be argued that we have not ruled out the possibility that the insertion of residues in the pre-M1 linker causes a rearrangement of the TMD that prevents the binding of allosteric ligands. If this were the case, then the lack of effect of TMD-binding ligands on the competition curves of the insertion mutants would have a trivial explanation that has nothing to do with domain−domain uncoupling. To test this possibility, we mutated the pore-lining leucine at position 9′ of the α7-AChR’s M2 α-helix (Fig. 1) to alanine. Mutations of this highly conserved leucine have long been known to increase the unliganded gating equilibrium constant of pLGICs (*29*, *45*–*47*) and thus are expected to shift the α-BgTx–nicotine competition curves to lower concentrations of the unlabeled ligand (*24*). Indeed, the half-competition concentration of nicotine decreased by a factor of 5.7 ± 0.50 (Fig. 6A and table S1). However, this effect of the L9′A mutation was smaller, or even altogether absent, when engineered in the background of mutants having extra residues in the pre-M1 linker, irrespective of whether the inserted residues were a single arginine (a decrease by a factor of 2.2 ± 0.35; Fig. 6B and table S1), a single alanine (a decrease by a factor of 1.3 ± 0.18; Fig. 6C and table S1), or two glycines (an increase by a factor of 1.1 ± 0.17; Fig. 6D and table S1). This result further supports the notion that, in pLGICs, the phenomenon of signal transduction is highly sensitive to the number of residues in the short stretch of amino acids covalently linking the ECD to the TMD.

### Naturally occurring insertions and deletions in pLGICs

**Fig. 7.**
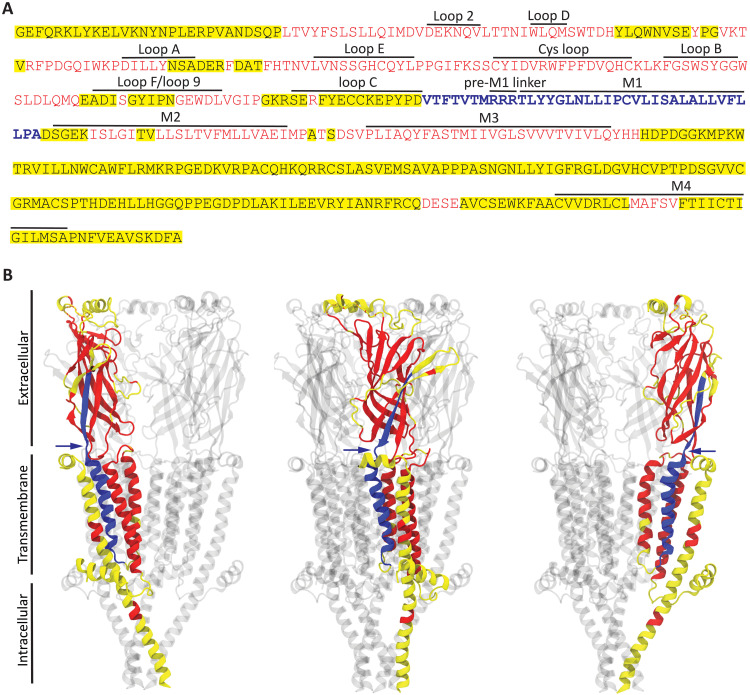
Naturally occurring insertions and deletions in pLGICs. (**A**) The sequences of 72 pLGIC-forming subunits—known, on the basis of electrophysiological recordings, to form fully functional receptor channels—were aligned using CLUSTAL W (*71*). Stretches of amino acids uninterrupted by indels in the entire set of 72 aligned sequences are indicated on the primary sequence of the human α7-AChR in red font, and the particular stretch that includes the pre-M1 linker is indicated in bolded-blue font. All other regions are indicated with a yellow background irrespective of the number of aligned sequences in which indels occur. The structural elements forming the orthosteric ligand-binding sites (loops A to F), those contributing to the extracellular side of the ECD−TMD interface (loop 2, Cys loop, and loop 9), the short stretch of amino acids covalently tethering the ECD to the TMD (the pre-M1 linker), and the four transmembrane segments (M1−M4) are indicated. The identities of the 72 aligned sequences are given in Materials and Methods. (**B**) The red-blue-yellow color code used in (A) is mapped onto three views of the atomic model of the unliganded (“apo”) human α7-AChR [PDB ID 7EKI (*72*)]. The blue arrows point to the pre-M1 linker. Note that the indel-permissive stretches (in yellow) flanking the length-invariant segment shown in blue occur in flexible regions of the protein—the loop C and the M1-M2 linker—where indels are likely to be accommodated without affecting the distance between the ECD and the TMD. The molecular image was made with VMD (*73*) using ribbon representation.

To learn about the conservation of the pre-M1 linker’s length in the pLGIC superfamily, we proceeded to align sequences from a diverse range of organisms. We restricted the alignment to sequences that code pLGIC subunits known, on the basis of electrophysiological recordings, to form fully functional receptor channels. The alignment contained a few sequences from bacteria, several sequences from invertebrates, and among vertebrates, it contained sequences only from human origin (see Materials and Methods). The alignment revealed that, although insertions and deletions (“indels”) of amino acids are pervasive and occur in functionally important regions of the protein (such as the extracellular loop A, Cys loop, loop F, and loop C; the transmembrane α-helices M2 and M4; and the M2−M3 linker), they spare a 37-residue segment that includes the pre-M1 linker (Fig. 7 and fig. S9). Evidently, whereas other regions of the channel have been able to accommodate amino-acid indels, leading, perhaps, to the emergence of novel functional properties, the length of the pre-M1 linker and flanking segments seems to have been much less tolerant to change. The notion that the length of this 37-residue segment has been highly conserved throughout evolution is consistent with the critical role ascribed here to the proximity between the ECD and the TMD in the binding–gating-coupling phenomenon of pLGICs.

## DISCUSSION

The phenomenon of domain–domain communication in multidomain proteins underlies not only the transduction of signals across membranes (*48*, *49*) but also the control of gene transcription (*50*), the enzyme-catalyzed synthesis of polyketides (*51*), the nonribosomal synthesis of peptides (*52*), the activity of AAA+ protein unfoldases (*53*), and the processivity of cytoskeletal motors (*54*), for example. In all of these cases, the length and flexibility of the interdomain linkers have been optimized for the specific functional needs of the modules they connect (*55*). In pLGICs, the five pre-M1 linkers ensure that the ECD and the TMD become functionally tied to each other, changing conformation as a unit. As can be inferred from the results presented here, such a tight coupling of the two domains is very likely the result of their close proximity, for which a short covalent linker seems ideally suited.

Electrically silent mutants occupy a special place in the field of mechanistic ion channel biology. Although experimentally challenging, the fact that these mutants point to regions of the protein that are uniquely critical for function makes them highly attractive targets of research. Perhaps, the best-known example of a highly informative electrically silent mutant is *Shaker*’s W434F (*56*). This mutant has maintained a constant presence in the literature of voltage-dependent K^+^ channels for decades (*57*–*61*) and has proven instrumental for the accurate measurement of gating currents (*62*, *63*) and the mechanistic characterization of the C-type inactivation phenomenon (*64*–*66*). Here, the study of electrically silent pLGICs allowed us to start unraveling the long-sought mechanism of ECD–TMD coupling in these ligand-gated ion channels.

Had it not been for the availability of quantitative methods to probe pLGIC function when measuring ion transport is not an option, our original identification of loss-of-current mutations at the ECD–TMD interface (*23*) could not have advanced beyond the level of a mere observation. Perhaps, we also would have tentatively ascribed the failure of these mutants to open upon agonist exposure to the uncoupling of the two domains for no reason other than the interfacial location of the mutated residues. Here, instead, using binding competition assays (*24*), we leveraged the effect of TMD-binding ligands and TMD mutations on the low-affinity ⇌ high-affinity conformational equilibrium of the ECD’s orthosteric sites to gain insight into the different mechanisms underlying the electrically silent phenotype. It is clear, now, that domain–domain uncoupling is not the only way for mutations at the interface to limit the opening of agonist-bound pLGICs. Indeed, it seems to us that the term “uncoupled” has been used somewhat loosely in the literature of these receptor channels. We propose that such a highly precise mechanistic term be used sparingly, exclusively to denote the effect of perturbations that, on the basis of functional assays, are found to weaken or eliminate the interdependence of the two domains.

Although the set of mutations analyzed here may, admittedly, be too small to make mechanistic generalizations, we wonder whether ECD–TMD coupling is such a robust property of the pLGIC modular architecture that the only type of mutation able to functionally uncouple the orthosteric ligand-binding sites from the transmembrane pore is that which physically separates the ECD from the TMD. Moreover, we wonder how mutations to structural elements outside the ECD–TMD interface (and far enough from the orthosteric sites as to not affect the latter’s ligand-binding affinities) would lead to the electrically silent phenotype. Last, we also wonder whether ECD–TMD coupling is an all-or-nothing phenomenon, and whether all five pre-M1 linkers are required to have a wild-type length for coupling to occur. The continued application of nonelectrophysiological methods to the functional characterization of loss-of-current mutations is poised to provide answers to these fundamental, but otherwise intractable, questions.

## MATERIALS AND METHODS

### cDNA clones and heterologous expression

cDNA coding the chimera between the ECD of the α7-AChR from chicken and the TMD of β-GluCl from *C. elegans* in pMT3 (*23*) was obtained by mutagenesis of a related clone provided by Y. Paas (Bar-Ilan University, Israel) (*10*). cDNA coding the human α7-AChR (UniProt accession number: P36544) in pcDNA3.1 was purchased from Addgene (no. 62276). cDNA coding isoform 1 of human RIC-3 (accession number: Q7Z5B4) (*67*) in pcDNA3.1 was provided by W. N. Green (University of Chicago, IL). cDNA coding human NACHO (TMEM35A; accession number: Q53FP2) (*68*) in pCMV6-XL5 was purchased from OriGene Technologies Inc. (no. SC112910). cDNA coding the human acid-sensing ion channel subunit 1 (ASIC1; accession number: P78348) in pCR-BluntII-TOPO was purchased from horizon (no. MHS6278-211689646) and was subcloned in pcDNA3.1. and cDNAs coding the mouse β1, δ, and ε subunits of the (muscle) AChR (accession numbers: P09690, P02716, and P20782, respectively) in pRBG4 were provided by S. M. Sine (Mayo Clinic, Rochester, MN). The chicken–*C. elegans* α7-AChR–β-GluCl chimera and the human α7-AChR were heterologously expressed in transiently transfected adherent HEK-293 cells grown at 37°C and 5% CO_2_. Transfections were performed using a calcium-phosphate precipitation method and proceeded for 16 to 18 hours after which the cell culture medium (Dulbecco’s modified Eagle’s medium; Gibco) containing the DNA precipitate was replaced by fresh medium. For the expression of the α7-AChR–β-GluCl chimera (for all types of experiments), we used 187.5 ng of cDNA/cm^2^. For the expression of the human α7-AChR for ligand-binding experiments, we used 125, 687.5, and 687.5 ng of cDNA/cm^2^ of cDNAs coding the human α7-AChR, human RIC-3, and human NACHO, respectively, whereas for electrophysiology experiments and the estimation of plasma membrane expression levels, we used one-fourth each of these amounts. As a control of the nonspecific binding of α-BgTx to transfected cells, HEK-293 cells were transiently transfected with cDNA coding human ASIC1 or the mouse β1-, δ-, and ε-AChR (but not α1-AChR) subunits. For the expression of human ASIC1, we used 187.5 ng of cDNA/cm^2^, whereas for expression of the mouse β1-, δ-, and ε-AChR subunits, we used 62.5 ng of cDNA/cm^2^ each. These mock-transfected cells were incubated with [^125^I]-α-BgTx (in the absence of unlabeled competitive ligand) under the same conditions as were the cells expressing the α7-AChR–β-GluCl chimera or the human α7-AChR. Mutations were engineered using the QuikChange kit (Agilent Technologies), and the sequences of the resulting cDNAs were verified by dideoxy sequencing of the entire coding region (ACGT).

### Ligand-binding curves

Twenty-four hours after changing the cell culture medium, transfected cells were resuspended in a Hepes-buffered sodium-saline solution (142 mM NaCl, 5.4 mM KCl, 1.8 mM CaCl_2_, 1.7 mM MgCl_2_, and 10 mM Hepes/NaOH, pH 7.4) by gentle agitation, and divided into 1-ml aliquots in 1.7-ml plastic tubes. Ligand-binding reaction mixtures were incubated at 37°C for 24 or 48 hours with constant rotation, and upon completion, cell-bound label was separated from unbound label by centrifugation at 16,000*g* for 3 min at room temperature. To reduce the amount of label bound nonspecifically to the cells, these pellets were resuspended in 1 ml of ice-cold Dulbecco’s phosphate-buffered saline (pH 7.4; Gibco), vortexed for 30 s, and pelleted again at 16,000*g* for 3 min at room temperature; this resuspension-pelleting procedure was repeated twice. Last, the washed pellets were solubilized in a solution containing 0.1 N NaOH and 1% (w/v) SDS, and incubated at 65° to 70°C for 30 min. The radioactivity and protein content of each solubilized pellet were estimated: ^125^I radioactivity was measured for 1 min using a Wiper 100 γ-counter (Laboratory Technologies Inc.), and the amount of protein was estimated using the bicinchoninic acid assay (Thermo Fisher Scientific) and a freshly prepared bovine serum albumin (Thermo Fisher Scientific) calibration curve. For any given construct, the number of transfected cells used in competition assays was adjusted, by trial and error, so as to minimize the depletion of labeled and unlabeled ligands while ensuring a sufficiently high signal. For some constructs, the expression of receptors was so high that the number of transfected cells that satisfied this criterion resulted in pellets that were too small to handle reliably. In these cases, transfected cells were mixed with nontransfected cells to increase the size of the pellets (*24*).

For all competition curves, the labeled ligand was [^125^I]-α-BgTx at a concentration (of the unbound form) of ~1 × *K*_D,closed_ at all values of unlabeled ligand concentration (fig. S2B). Toxin *K*_D,closed_ values for the α7-AChR–β-GluCl chimera, the M2−M3 linker-replacement mutant, the F157A mutant, and the chimera’s arginine and double-glycine insertion mutants, as well as the full-length human α7-AChR, were estimated from saturation curves at equilibrium (fig. S3, A to E, and G) as previously described (*24*). For all other mutants, however, their lower levels of expression rendered the corresponding saturation curves inconclusive. At the high concentrations of toxin needed to saturate the receptor (>10 nM), the nonspecific binding of α-BgTx to the cell surface of HEK-293 cells obscured the amount of specific binding to the relatively few receptor sites on the plasma membrane, even after profuse washes. Thus, for this subset of constructs, we resorted to a different method: We performed binding competition assays between labeled toxin (present at a fixed concentration) and unlabeled toxin. Although this method is more approximate and less direct than a saturation assay, it works best with nonsaturating concentrations of label, which in turn results in less nonspecific binding. Because the binding of α-BgTx favors the receptor’s closed-channel conformation (*29*–*31*)—and because, in these competition assays, the receptor transitions from being (partially) bound to labeled toxin to being (fully) bound to unlabeled toxin—the receptor is expected to remain closed throughout the curve. Under these particular conditions, it can be shown that the mean number of binding sites occupied by labeled toxin (*N*) is given by

$$N=\frac{n\times \left[\text{Labeled toxin}\right]}{K_{D,\text{closed}}+\left[\text{Labeled toxin}\right]+\left[\text{Unlabeled toxin}\right]}$$ (2)

where, *n* denotes the total number of toxin-binding sites per receptor, the concentration of labeled toxin is fixed, the concentration of unlabeled toxin is the variable of the assay, and *K*_D,closed_ denotes the dissociation equilibrium constant of the toxin from the receptor’s closed-channel conformation. It follows, then, that the concentration of unlabeled toxin required to half-compete the binding of labeled toxin is given by

$$\left[\text{Half-competition}\right]=K_{D,\text{closed}}+\left[\text{Labeled toxin}\right]$$ (3)

from where an estimate of *K*_D,closed_ can be calculated for any given value of the concentration of labeled toxin and the experimentally estimated value of the half-competition concentration. These *K*_D,closed_ estimates and the corresponding competition curves are shown in fig. S3 (F and H to J). Consistent with the notion that none of the mutations reported in this study occurred at positions predicted to affect the affinity of the α7-AChR for α-BgTx (*69*, *70*), all experimentally determined values of toxin affinities for the mutants were close to their wild-type counterparts’.

For each individual binding competition curve (whether toxin–toxin or toxin–small-molecule), each concentration of unlabeled ligand was assayed in duplicate. Individual curves generated using the same combination of construct and orthosteric-ligand/TMD perturbation were globally fitted with Hill equations, and SEs of the fitted parameters were computed using the reduced χ statistic in weighted regression using SigmaPlot 14 or 15 (Systat Software Products). The number of required Hill equation components in the fits was assessed on the basis of goodness-of-fit statistics and the relative size of the errors; all competition curves discussed in this work were best fitted with single-component Hill equations. The number of independent competition curves contributing to each globally fitted curve is indicated in table S1; errors of the individual datapoints were calculated only when the latter was larger than 2. Error bars (±1 SE) smaller than the size of the symbols were omitted. [I]-α-BgTx was purchased from Revvity, unlabeled α-BgTx from Alomone Labs, MLA and PNU-120596 from Tocris Bioscience, and ACh, carbamylcholine, nicotine, TMA, IVM, etomidate, propofol, picrotoxinin, lindane, and menthol, from MilliporeSigma. IVM, etomidate, propofol, picrotoxinin, lindane, menthol, and PNU-120596 were dissolved in dimethyl sulfoxide (DMSO), and the final concentration of the latter in the working solutions was 0.1% (v/v). The negligible effect of DMSO at this concentration on the α-BgTx–nicotine binding curves is shown in fig. S5.

### Plasma membrane expression

The number of receptors on the plasma membrane of transfected HEK-293 cells was estimated from the amount of [^125^I]-α-BgTx bound upon incubation with a saturating concentration of toxin (~30 nM) for 4 hours at 4°C, as previously described (*23*). The low temperature was intended to minimize the uptake of toxin through endocytosis. Once the incubation was completed, the amount of radioactivity specifically bound to plasma membrane receptors was quantified following the same steps as described above for the binding competition curves. Because this type of assay reports on expression levels averaged over all cells in a sample, and because plasma membrane expression levels vary widely from cell to cell, the relationship between the reported levels of expression and the size of the peak currents recorded from individual cells is usually not linear. This is especially true when (as was the case here) only cells that looked well transfected were “patched.” Thus, in our hands [this work and (*23*)], mutant–to–wild type expression level ratios higher than 0.01 turned out to be high enough for robust currents to be recorded from functional mutants in at least some of the patched cells. Only expression level ratios lower than 0.01 were considered to be too low. All of the constructs deemed here to be electrically silent expressed above this threshold.

### Electrophysiology

Currents were recorded in the whole-cell configuration of the patch-clamp technique at ~22°C with an effective bandwidth of DC–5 kHz using an Axopatch 200B amplifier (Molecular Devices). Currents were digitized at 100 kHz and analyzed using pCLAMP 11.1 software (Molecular Devices). Series resistance compensation was used and set to ~80%. The reference Ag/AgCl wire was connected to the extracellular solution through an agar bridge containing 200 mM KCl. Agonist concentration jumps were applied to whole cells using a piece of double-barreled “θ-tubing” (Siskiyou). The flow of solution through the θ-tube was controlled using a gravity-fed system (ALA BPS-8; ALA Scientific Instruments), and the movement of the θ-tube was achieved using a piezoelectric arm (Burleigh-LSS-3100; discontinued) controlled by pCLAMP 11.1 software (Molecular Devices). The latter signals were low-pass filtered (900C; Frequency Devices) at a cutoff frequency of ~25 to 30 Hz before their arrival at the piezoelectric arm to reduce ringing in the θ-tube motion. During experiments, patched cells remained attached to a piece of collagen-coated glass coverslip (Neuvitro) placed at the bottom of the recording chamber. In this particular configuration, the perfusion system achieved a solution exchange time of ~4 ms for the *t*_10–90%_ and ~10 ms for the *t*_90–10%_, as estimated from changes in the liquid junction current measured with an open-tip patch pipette. For all recordings, the pipette solution was 110 mM KCl, 40 mM KF, and 5 mM HEPES/KOH (pH 7.4). For recordings from cells expressing the α7-AChR–β-GluCl chimera and its mutants (with the exception of the NWCL mutant; fig. S1), the extracellular solution (flowing through the two barrels of a piece of θ-tubing) was 15 mM KCl, 230 mM mannitol, and 5 mM HEPES/KOH (pH 7.4), with or without 100 μM ACh; the bath solution was extracellular solution without ACh. For recordings from cells expressing the NWCL mutant of the α7-AChR–β-GluCl chimera (fig. S1), the extracellular solution was 5 mM KCl, 250 mM mannitol, and 5 mM HEPES/KOH (pH 7.4), with or without 100 μM ACh; the bath solution was extracellular solution without ACh. For recordings from cells expressing the human α7-AChR and its mutants, the extracellular solution was 142 mM NaCl, 5.4 mM KCl, 1.8 mM CaCl_2_, 1.7 mM MgCl_2_, and 10 mM HEPES/NaOH (pH 7.4), with or without 100 μM ACh; the bath solution was extracellular solution without ACh. Patch pipettes, pulled from thin-walled borosilicate glass capillary tubing (Sutter Instrument), had resistances of 3 to 5 megohms when filled with pipette solution.

### Sequence alignment

The sequences of 72 pLGIC-forming subunits—known, on the basis of electrophysiological recordings, to form fully functional receptor channels—were aligned using CLUSTAL W with default parameters (*71*). Forty-one of these 72 sequences code for the following human subunits: α1–7-, α9-, α10-, β1–4-, δ-, γ-, and ε- AChRs; α1–3- and β- glycine receptors; serotonin types 3A and 3B receptors; α1–6-, β1–3-, γ1–3-, δ-, ε-, π-, and ρ1–3- GABA_A_Rs; and the zinc-activated channel. Twenty-seven sequences code for the following invertebrate subunits: LnAChR_A and B from *Lymnaea stagnalis*; SmACC-1 from *Schistosoma mansoni*; ACR-23, GluCl α1, GluCl β, EXP-1, LGC-35, LGC-40, LGC-41, LGC-53, LGC-55, MOD-1, ACC-1–4, and PBO-5 and 6 from *C. elegans*; Hco_ACC-2 from *Haemonchus contortus*; UNC-29 and 38 from *Ascaris suum*; and RDL, GRD, LCCH3, and HisCl α1 and 2 from *Drosophila melanogaster*. In addition, four sequences code for the following bacterial subunits: GLIC from *Gloeobacter violaceus*; ELIC from *Dickeya dadantii*; sTeLIC from *Tevnia jerichonana*; and DeCLIC from a *Desulfofustis* deltaproteobacterium.

The alignment was analyzed in terms of frequency of deletions and insertions relative to the sequence of the human α7-AChR (fig. S9). To this end, the entire alignment was “cut” so as to start with the first residue in the mature sequence of the α7-AChR (Gly^23^) and end with the last residue (Ala^502^). Likening this subset of the alignment to a matrix, the latter contained 72 rows (that is, the number of aligned sequences) and 927 columns (that is, the number of positions in the alignment required to accommodate the gaps). Columns with 72 elements were assigned a value of zero frequency of deletion or insertion. Columns with fewer than 72 elements were classified as containing deletions if the row corresponding to the α7-AChR contained an element (that is, if the missing elements corresponded to rows other than the α7-AChR’s). Conversely, columns with fewer than 72 elements were classified as containing insertions if the row corresponding to the α7-AChR did not contain an element. The frequency of deletions was calculated for each column as (72 − *x*)/72, where *x* is the number of elements in the corresponding column. Because the lowest possible value of *x* is 1, the maximum value of the deletion frequency is 71/72 (=0.986). This would be the case for columns at which only the α7-AChR contains an amino acid, and thus, the frequency of deletion (from the perspective of the α7-AChR) would be high. The frequency of insertions was calculated for each column as *x*/72, where *x* is the number of elements in the column. The highest possible value of *x* is 71, and hence, the maximum value of the insertion frequency is 0.986. This would be the case for columns at which every row, with the exception of the α7-AChR’s, contains an amino acid, and thus, the frequency of insertion would be high.
