# Supplementary material for: Electrically silent mutants unravel the mechanism of binding−gating coupling in Cys-loop receptors
Source: Sci Adv. 2024 Nov 27;10(48):eadq8048. doi: 10.1126/sciadv.adq8048 (PMC11601209; doi:10.1126/sciadv.adq8048)
Supplement: Supplementary file 1 — Figs. S1 to S9 Tables S1 and S2 [file sciadv.adq8048_sm.pdf]

Science Advances
AAAS

Supplementary Materials for

Electrically silent mutants unravel the mechanism of binding-gating coupling in Cys-loop receptors

Nicole E. Godellas *et al.*

Corresponding author: Claudio Grosman, grosman@illinois.edu

Sci. Adv. **10**, eadq8048 (2024)
DOI: 10.1126/sciadv.adq8048

This PDF file includes:

Figs. S1 to S9
Tables S1 and S2

Fig. S1.

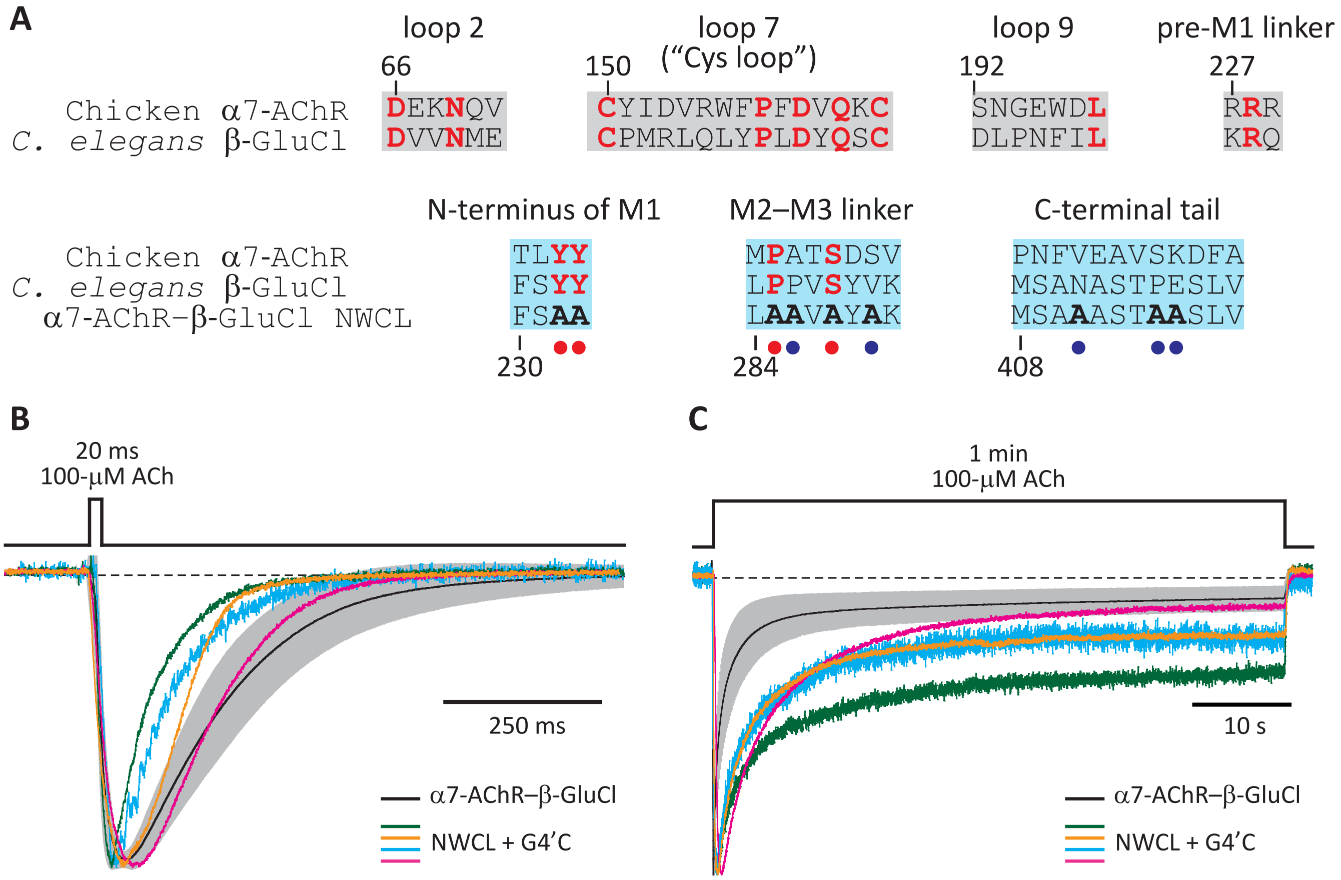

The α7-AChR–β-GluCl ECD–TMD chimera and the NWCL mutant. (**A**) Sequence alignment of the structural elements that form the ECD–TMD interface of all pLGICs. A different-color background indicates whether these stretches of amino acids map to the ECD (gray) or the TMD (cyan). Residues conserved in both parental sequences are indicated with red single-letter amino-acid symbols. To obtain the NWCL mutant ("No Wild-type Contact Left"), the four side chains on the (β-GluCl) TMD side of the domain–domain interface that are conserved in the α7-AChR were mutated to alanine (red circles). Although not strictly conserved, five other β-GluCl residues were also mutated to alanine (blue circles), for the sake of thoroughness. Thus, the NWCL construct contains two mutations in the N-terminus of M1 (Y232A and Y233A), four mutations in the M2–M3 linker (P285A, P286A, S288A, and V290A), and three mutations in the C-terminal tail (N411A, P415A, and E416A) for a total of 45 mutations in the TMD side of the pentameric interface. Because the four mutations in the M2–M3 linker slow down the chimera's desensitization time course (*23*), the G266C mutation (at position 4′ of the M2 pore-lining α-helix, near the cytosolic end of the pore, and ~30-Å away from the domain–domain interface), which speeds up desensitization (*23*), was also engineered. (**B** and **C**) Normalized inward currents recorded from the indicated constructs in the whole-cell configuration. For the "wild-type" α7-AChR–β-GluCl chimera, responses are shown as the mean (black solid line) ± one standard deviation (SD; gray error bars) of responses recorded from different whole-cell experiments. Wild-type data in *B* correspond to a total of 282 responses recorded from 35 different cells. Wild-type data in *C* correspond to a total of 39 responses recorded from 30 different cells. The membrane potential was ~–60 mV. Black dashed lines denote the zero-current baseline. In previous work (*23*), each structural element of the ECD–TMD interface was mutated individually. Here, all three elements on the TMD side were mutated together. Notably, despite the lack of sequence conservation across the ECD–TMD interface, the mutant bearing the NWCL mutations responded to short and long ACh-concentration pulses in a manner that is characteristic of naturally occurring members of the superfamily. The different levels of noise of the displayed current traces reflect the different values of the peak-current amplitude recorded in the different cells.

Fig. S2.

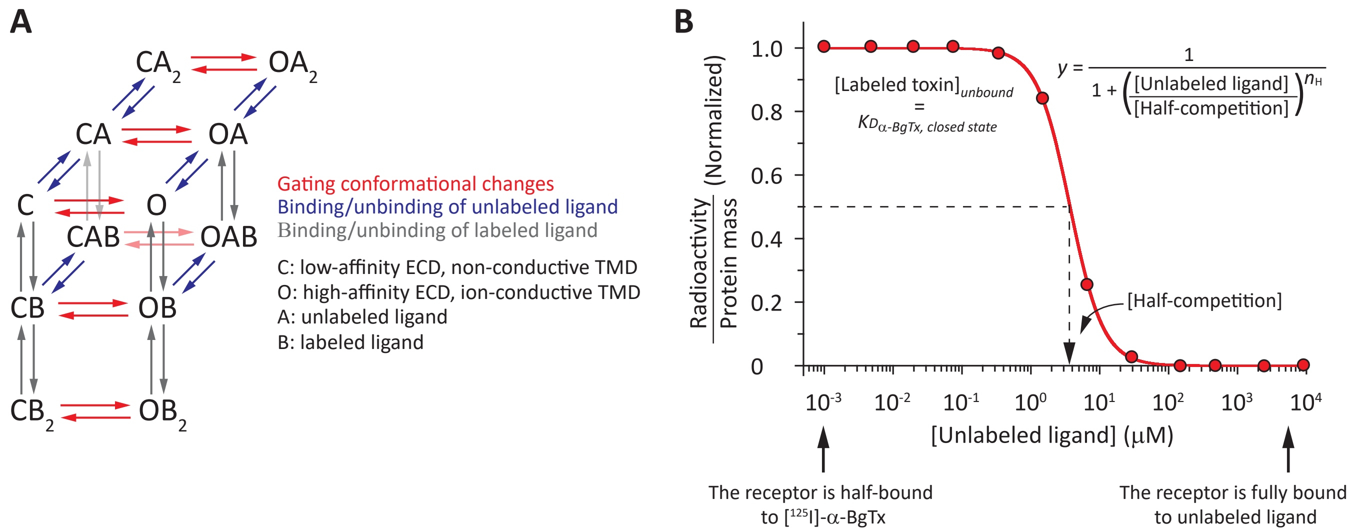

Probing function in pLGICs when measuring ion transport is not an option. (**A**) A kinetic scheme that encapsulates our interpretation of the phenomena at play during our binding-competition assays between labeled and unlabeled orthosteric ligands. For clarity of display, the scheme only shows two of the five orthosteric sites of the α7-AChR's ECD. Also, for the sake of simplicity, the open and desensitized conformations—that is, the conformations of the receptor-channel that bind neurotransmitter and other agonists with higher affinity—were grouped together in a single "O" state. (**B**) Schematic representation of a binding-competition curve. The data points and the solid line were calculated and are only meant to illustrate the properties of a typical curve. In our binding-competition assays, the concentration of unbound ("free") [^{125}I]-α-BgTx at equilibrium was kept approximately constant and equal to its dissociation equilibrium constant (K_D) from the closed-channel conformation ($K_{D,closed}$) at all values of unlabeled-ligand concentration (as inferred from the radioactivity present in the reactions' supernatants at the end of the 24- or 48-h incubations (*24*)). Hence, the mean number of binding sites occupied by the labeled toxin in the absence of competing unlabeled ligand was, approximately, one-half of the total α-BgTx-binding sites (that is, ~2.5 per receptor). The concentration of competing unlabeled ligand that lowers the latter by a factor of 2 is denoted, here, as the "half-competition concentration". Throughout this paper, the concentration of unlabeled ligand plotted on the curves' *x*-axes corresponds to the total (bound plus unbound) concentration. Under the low ligand-depletion conditions of our experiments, this concentration was deemed to be a good approximation for the concentration of unbound unlabeled ligand at equilibrium (*24*). In the absence of competing unlabeled ligand (that is, at the leftmost end of the curve), the α7-AChR predominantly occupies the closed state. When the competing ligand is an agonist, the conformation of the orthosteric sites changes from the low-affinity conformation to the high-affinity conformation as the concentration of unlabeled ligand increases and α-BgTx is outcompeted. That is, in going from one end of the curve to the other, agonists bind to different conformations of the receptor. This explains why the half-competition concentration of nicotine depends not only on its low- and high-affinity K_Ds, but also, on the receptor's gating equilibrium constants. It can be shown that, in the case of a receptor with five binding sites, the mathematical expression for the half-competition concentration of an agonist is particularly unwieldy. In marked contrast, when the unlabeled ligand competing against α-BgTx is an inverse agonist, the conformation of the orthosteric sites remains unchanged throughout the curve. This explains why the half-competition concentration of MLA is such a mathematically straightforward indicator of its K_D from the closed state. The one-component Hill equation is shown. n_H = Hill coefficient. [Half-competition] = half-competition concentration.

Fig. S3.

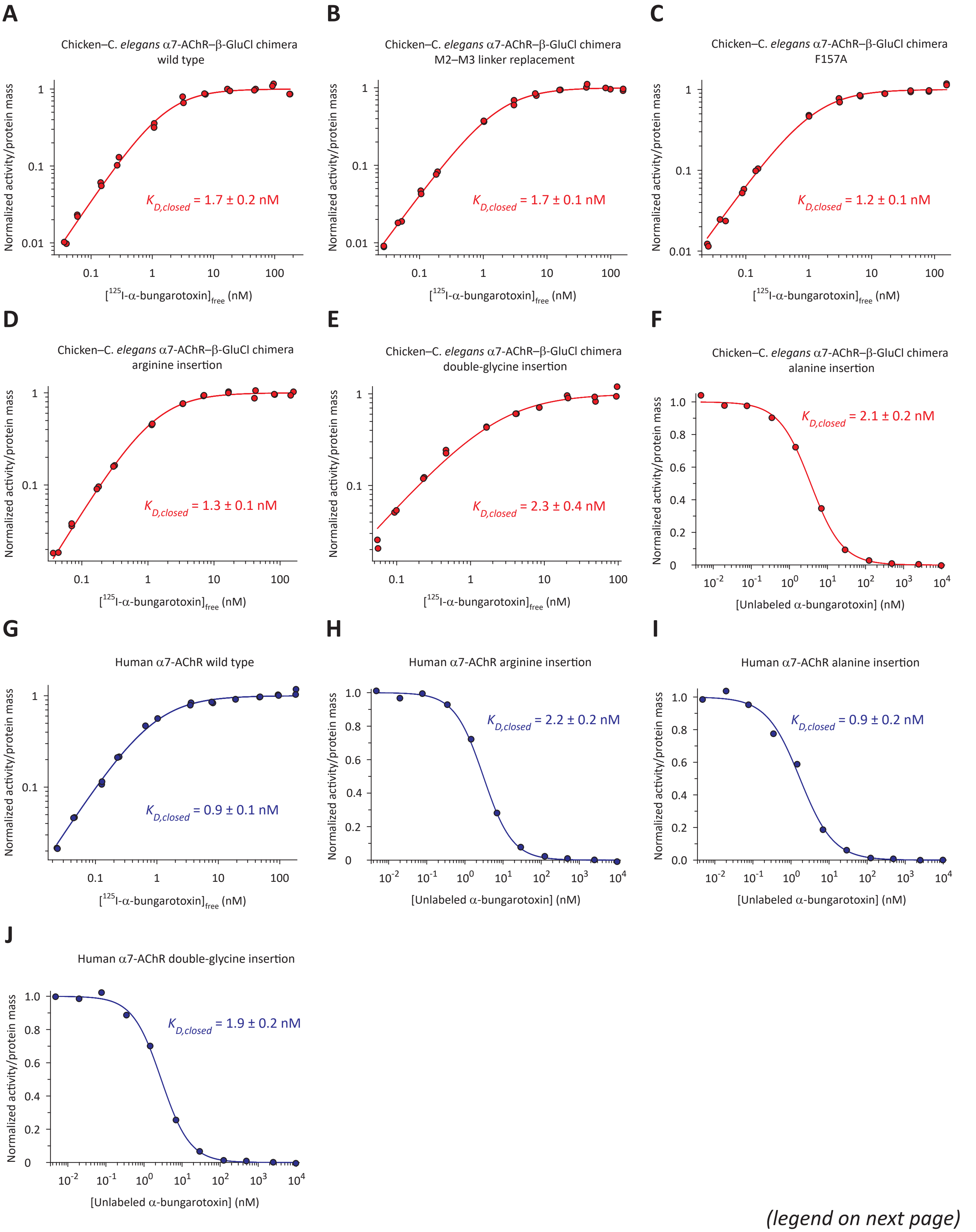

(legend on next page)

α-BgTx affinities for the different constructs studied in this work. (A to **F)** α7-AChR–β-GluCl chimera wild-type (*24*) and mutants. (**G** to **J**) α7-AChR (full-length) wild-type (*24*) and mutants. $K_{D,closed}$ values of α-BgTx were estimated from toxin-saturation curves (as the half-saturation concentration of fits with a single-component Hill-equation) or from competition curves between labeled and unlabeled toxin (solving Eq. 3; see Materials and Methods). The errors of the half-saturation and half-competition concentration estimates were computed as part of the curve-fitting procedure. In the competition assays, the (measured) concentration of unbound [^{125}I]-α-BgTx was ~1.5 nM (panel **F**) or ~0.9 nM (panels **H** to **J**). Saturation-assay reactions were incubated for 24 h, whereas competition-assay reactions were incubated for 24 or 48 h.

Fig. S4.

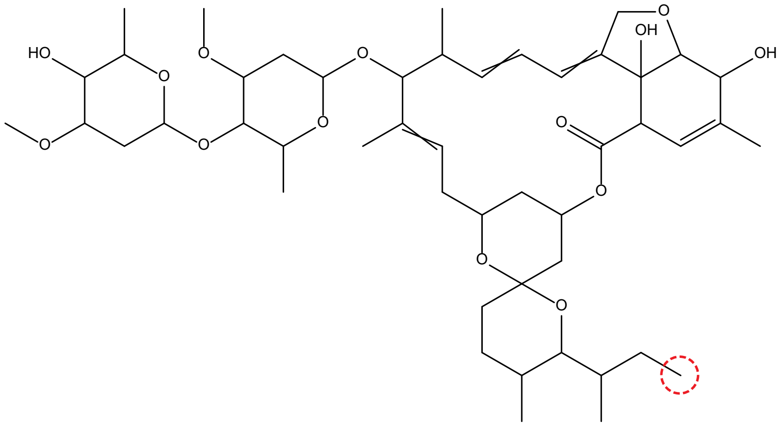

Ivermectin B1a
(>80%)

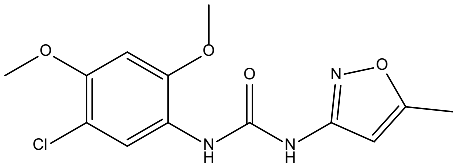

PNU-120596

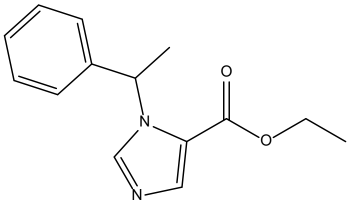

Etomidate

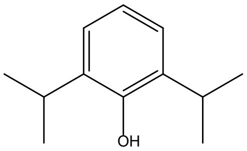

Propofol

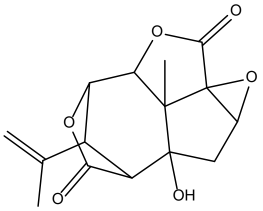

Picrotoxinin

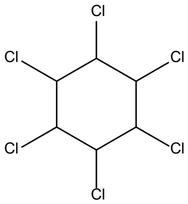

Lindane

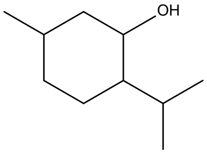

Menthol

Structures of the TMD-binding ligands used in this work. Ivermectin (IVM) is a mixture of two compounds, IVM B1a (>80%) and IVM B1b. In the latter, the methyl group indicated on IVM B1a with a red circle is replaced by a hydrogen atom.

Fig. S5.

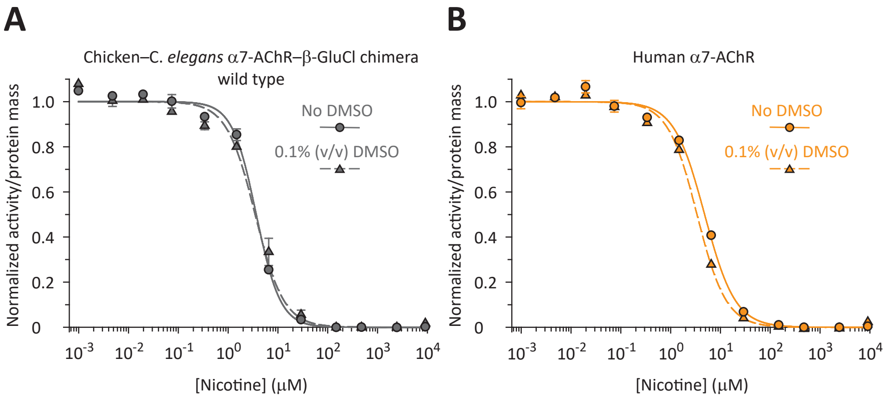

Effect of DMSO on α-BgTx–nicotine binding-competition curves. (**A**) Chicken–*C. elegans* α7-AChR–β-GluCl chimera. (**B**) Human α7-AChR. All TMD-binding ligands tested in this work were dissolved in DMSO in such a way that its final concentration in the competition reaction mixtures was 0.1% (v/v). The curves were fitted with single-component Hill equations (table S1), and for all of them, the labeled ligand was [^{125}I]-α-BgTx at a concentration (of the unbound form) of ~1×$K_{D,closed}$ (fig. S3). At the concentration of DMSO employed here, we deemed its effect on the binding-competition curves to be negligible.

Fig. S6.

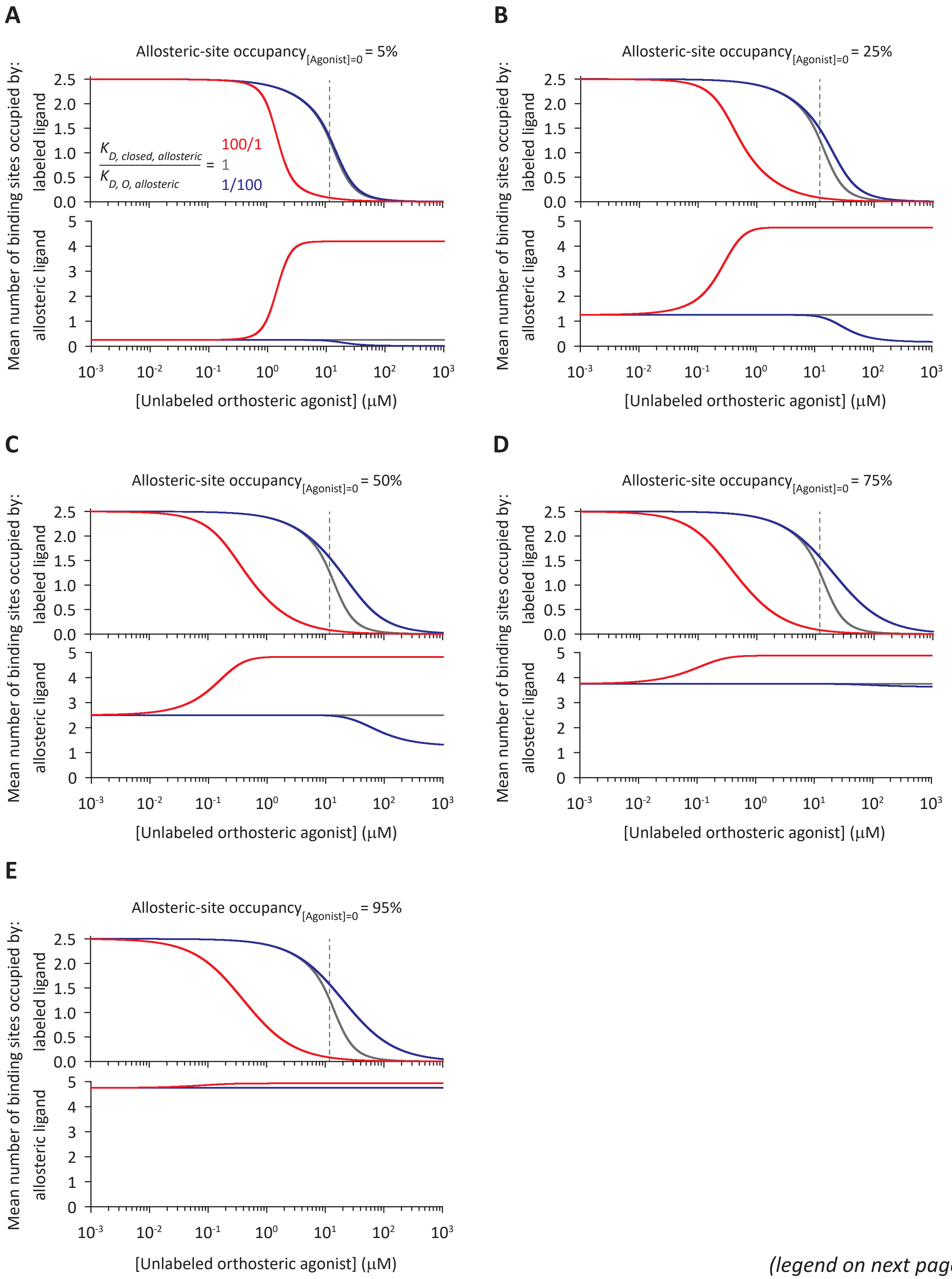

(legend on next page)

Effect of state-stabilizing allosteric ligands on the competition between labeled and unlabeled orthosteric ligands. To illustrate the effect of state-stabilizing allosteric ligands on the binding-competition curves for orthosteric ligands, we performed calculations in the framework of the kinetic model schematically shown in Figure S2A (see also ref. (*24*)) with the addition of five allosteric ligand-binding sites (one per subunit). Therefore, the modeled receptor-channel interacts with three different ligands: two orthosteric ligands (one labeled and one unlabeled competing for the same sites) and one allosteric ligand. Moreover, each of these ligands binds with distinct affinities to the different conformations of the receptor-channel, and hence, stabilizes them relative to each other accordingly. On the basis of experimental evidence (*24*), the five orthosteric sites were considered to be identical and independent of each other's occupancy. In the absence of more detailed information, the five allosteric sites were assumed to be identical and independent, as well. Moreover, orthosteric ligands were assumed to bind to the open and desensitized conformations with indistinguishable affinities, and merely for the sake of simplicity, the same was assumed to be the case for the particular allosteric ligands modeled here. As a result, the open and desensitized conformations were grouped together in a single "O" state (fig. S2A), thus reducing the total number of ligation/conformation states in the model from 378 to 252. Each panel shows the expected effect of three allosteric ligands that differ in the ratio of their affinities for the closed state *versus* the O (open + desensitized) states: illustrated in red is a positive allosteric modulator; in blue, a negative allosteric modulator; and in gray, an antagonist. In going from **A** to **E**, the different panels illustrate the effect of increasing the concentration of allosteric ligand (a constant value throughout each curve). For any given panel, these concentrations were adjusted in such a way that each curve starts with the same mean number of allosteric-ligand molecules bound per receptor. The depletion of ligands by binding to the receptor was assumed to be negligible, and thus, total and free concentrations are indistinguishable. The modeled labeled orthosteric ligand was an antagonist with a dissociation equilibrium constant (K_D) from closed and O states of 1 nM; its concentration was kept constant at $1\times K_D$ for all points of the curves, that is, at a concentration that half-saturates the receptor in the absence of competing unlabeled orthosteric ligand. The unlabeled orthosteric ligand was an agonist with a K_D from the closed state of 10 μM and a K_D from the O state of 200 nM. The allosteric ligand had a K_D from the closed state of 1 μM and a K_D from the O state given by the indicated ratios, namely, 10 nM (red curves), 1 μM (gray curves) or 100 μM (blue curves). The unliganded closed $\rightleftharpoons$ O gating equilibrium constant was set to 10^{-7}. As the concentration of allosteric ligand rose (that is, in going from panel **A** to panel **E**), the binding-competition curves moved increasingly to the right, for the negative allosteric modulator (blue curves); to the left, for the positive allosteric modulator (red curves); and stayed unaffected, for the antagonist (gray curves). Note that, unless the allosteric ligand binds with the same affinity to closed and O states (gray curves), the occupancy of the allosteric sites by positive or negative modulators depends on the concentration of orthosteric agonist. In some panels, the plots in blue and gray completely overlap. As a reference, the half-competition concentration of unlabeled orthosteric agonist in the presence of an allosteric antagonist (gray curves) is indicated in each upper panel with a vertical gray dashed line.

Fig. S7.

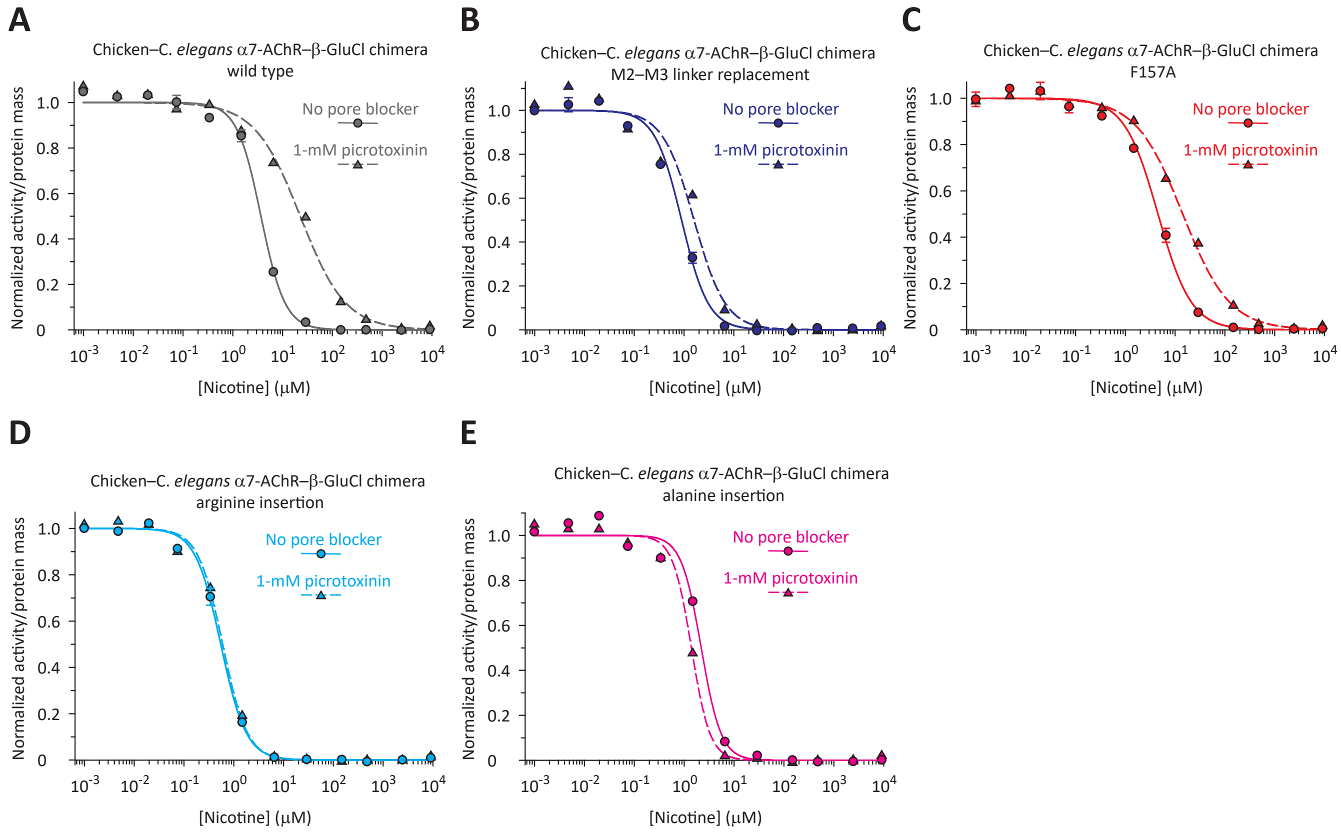

Probing pore conformation in the α7-AChR–β-GluCl chimera using the open-channel blocker picrotoxinin. (**A** to **C**) Well-coupled constructs. (**D** and **E**) Negative controls using ECD–TMD-uncoupled mutants. The curves were fitted with single-component Hill equations (table S1), and for all of them, the labeled ligand was $[^{125}I]$-α-BgTx at a concentration (of the unbound form) of ~1×$K_{D,closed}$ (fig. S3).

Fig. S8.

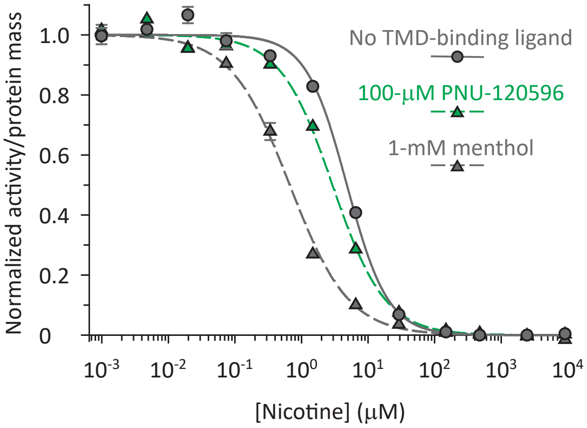

Effects of menthol and PNU-120596 on the α-BgTx–nicotine competition curves of the human α7-AChR. Whereas 1-mM menthol decreased the half-competition concentration of nicotine by a factor of 7.3 ± 0.58, 100-μM PNU-120596 decreased it by a factor of 1.7 ± 0.14. The curves were fitted with single-component Hill equations, and for all of them, the labeled ligand was [^{125}I]-α-BgTx at a concentration (of the unbound form) of ~1×$K_{D,closed}$ (fig. S3).

Fig. S9.

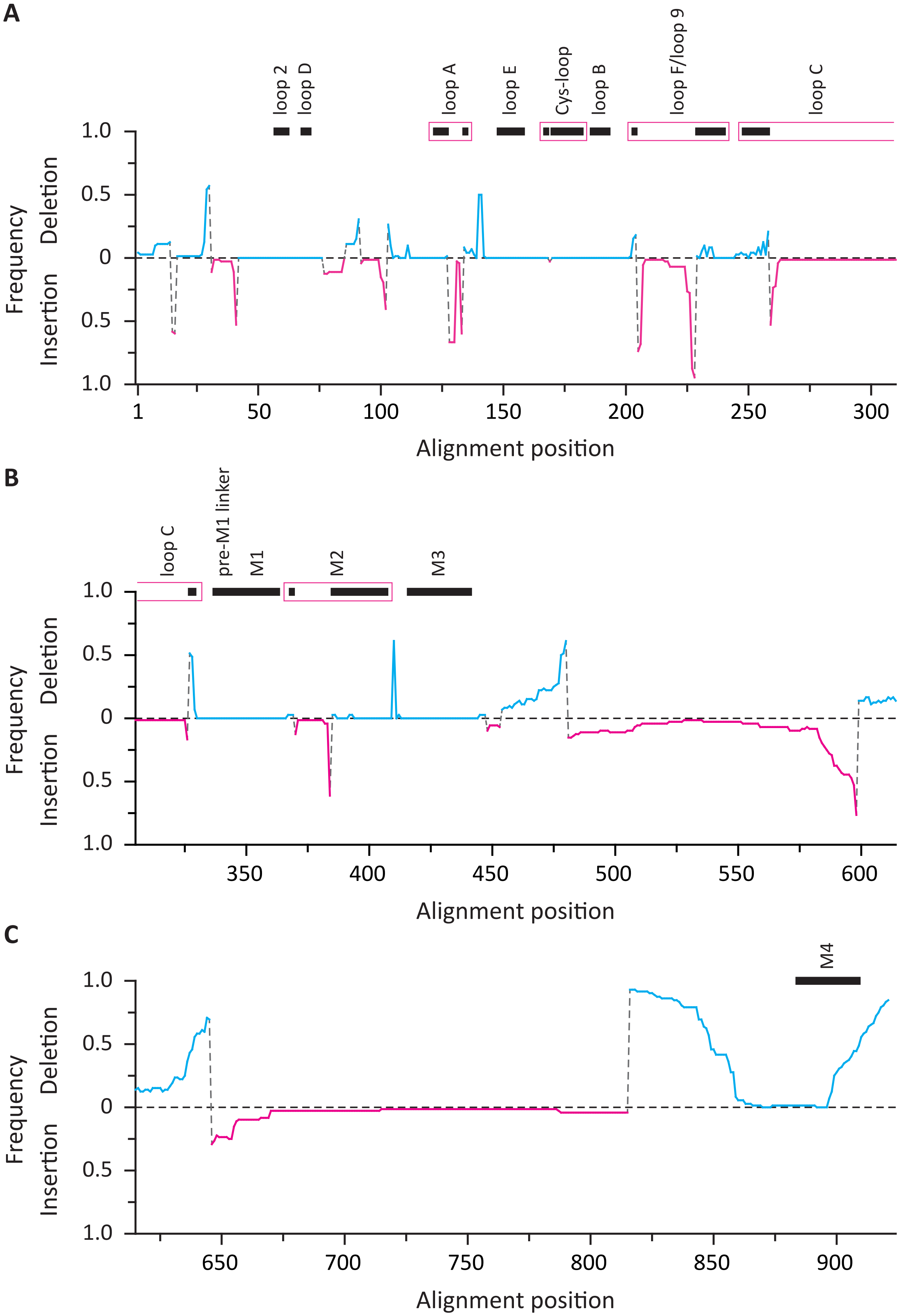

(legend on next page)

Quantitative analysis of insertions and deletions in pLGICs. The sequences of 72 pLGIC-forming subunits—known, on the basis of electrophysiological recordings, to form fully functional receptor-channels—were aligned, and the resulting alignment was analyzed in terms of frequency of deletions or insertions relative to the sequence of the human α7-AChR (see also Fig. 7). Because of the occurrence of insertions, a total of 927 positions were required in the alignment to accommodate the 480 residues of the α7-AChR. For the sake of clarity, the results are shown in three panels (**A** to **C**) corresponding to consecutive sections of the alignment. Deletion and insertion frequencies were calculated as indicated in Materials and Methods. Stretches of consecutive zeroes represent segments of amino-acid residues uninterrupted by insertions or deletions in the entire set of 72 sequences; one such stretches is the 37-residue segment that includes the pre-M1 linker. Alignment positions containing α7-AChR residues are denoted in cyan, and those at which α7-AChR residues are missing are denoted in magenta. A black horizontal dashed line indicates the zero "baseline", and gray dashed lines connect discontinuous regions of the plot (that is, cyan → magenta and magenta → cyan "jumps"), merely for clarity. Various structural elements (orthosteric-site loops A–F; loop 2, Cys loop, loop 9, and pre-M1 linker at the ECD–TMD interface; and transmembrane segments M1–M4) are mapped onto the sequence of the human α7-AChR with horizontal black bars. Broken bars bound together by magenta rectangles denote the occurrence of gaps in the alignment as a result of insertions.

Table S1.

Construct	Unlabeled ligand	TMD perturbation	Unlabeled-ligand [Half-competition]	Unlabeled-ligand Hill coefficient	*n*
Chicken–C. *elegans* α7-AChR–β-GluCl ECD–TMD chimera	Carbamylcholine	—	1.50 ± 0.10 mM	1.70 ± 0.13	2
	Nicotine	—	3.68 ± 0.11 μM	1.80 ± 0.07	3
	Nicotine	0.1% (v/v) DMSO	3.50 ± 0.27 μM	1.51 ± 0.12	3
	Nicotine	100-μM ivermectin	23.5 ± 1.91 μM	1.28 ± 0.09	2
	Nicotine	100-μM etomidate	10.9 ± 0.87 μM	1.35 ± 0.10	2
	Nicotine	100-μM propofol	9.43 ± 0.99 μM	1.45 ± 0.16	2
	Nicotine	1-mM picrotoxinin	22.7 ± 2.64 μM	0.95 ± 0.06	2
	Nicotine	1-mM lindane	22.1 ± 3.06 μM	1.00 ± 0.10	2
	TMA	—	620 ± 33 μM	1.76 ± 0.11	2
	TMA	100-μM ivermectin	2,550 ± 200 μM	1.47 ± 0.11	2
	MLA	—	4.90 ± 0.51 nM	0.99 ± 0.07	3
	MLA	100-μM ivermectin	6.33 ± 0.82 nM	0.80 ± 0.07	2
	MLA	100-μM etomidate	4.03 ± 0.27 nM	0.91 ± 0.04	2
M2–M3 linker replacement*	Carbamylcholine	—	0.27 ± 0.01 mM	1.62 ± 0.07	2
	Nicotine	—	0.89 ± 0.06 μM	1.61 ± 0.10	3
	Nicotine	100-μM ivermectin	11.3 ± 0.82 μM	1.33 ± 0.10	3
	Nicotine	1-mM picrotoxinin	1.59 ± 0.21 μM	1.37 ± 0.17	2
	TMA	—	105 ± 6.31 μM	1.46 ± 0.09	2
	MLA	—	5.18 ± 1.03 nM	1.02 ± 0.16	2
F157A†	Carbamylcholine	—	1.74 ± 0.08 mM	1.25 ± 0.04	2
	Nicotine	—	4.53 ± 0.25 μM	1.29 ± 0.06	3
	Nicotine	100-μM ivermectin	41.6 ± 2.87 μM	1.14 ± 0.06	2
	Nicotine	1-mM picrotoxinin	14.2 ± 1.15 μM	0.92 ± 0.05	2
	TMA	—	845 ± 76 μM	1.09 ± 0.07	2
	MLA	—	5.07 ± 0.52 nM	0.97 ± 0.07	2
Arginine insertion‡	Carbamylcholine	—	0.21 ± 0.01 mM	2.00 ± 0.13	2
	Nicotine	—	0.55 ± 0.02 μM	1.70 ± 0.08	3
	Nicotine	100-μM ivermectin	0.95 ± 0.05 μM	2.00 ± 0.18	2
	Nicotine	1-mM picrotoxinin	0.60 ± 0.04 μM	1.75 ± 0.14	2
	TMA	—	103 ± 6.22 μM	2.15 ± 0.19	2
	MLA	—	3.83 ± 0.37 nM	0.99 ± 0.07	2
Alanine insertion‡	Nicotine	—	2.19 ± 0.16 μM	2.17 ± 0.19	2
	Nicotine	100-μM ivermectin	1.75 ± 0.06 μM	2.31 ± 0.16	2
	Nicotine	1-mM picrotoxinin	1.39 ± 0.07 μM	2.32 ± 0.27	2
Double-glycine insertion‡	Nicotine	—	4.04 ± 0.29 μM	1.22 ± 0.07	2
	Nicotine	100-μM ivermectin	3.42 ± 0.22 μM	1.55 ± 0.11	2
Human α7-AChR wild type	Nicotine	—	4.82 ± 0.24 μM	1.41 ± 0.06	3
	Nicotine	0.1% (v/v) DMSO	3.39 ± 0.23 μM	1.48 ± 0.10	2
	Nicotine	1-mM menthol	0.66 ± 0.04 μM	0.98 ± 0.04	4
	Nicotine	100-μM PNU-120596	2.92 ± 0.20 μM	1.1 ± 0.06	1
	Nicotine	L9′A	0.85 ± 0.06 μM	0.91 ± 0.04	2
	MLA	—	88.9 ± 8.54 nM	0.95 ± 0.05	5
	MLA	1-mM menthol	73.4 ± 5.54 nM	0.91 ± 0.04	2
Arginine insertion‡	Nicotine	—	2.04 ± 0.29 μM	0.79 ± 0.06	2
	Nicotine	1-mM menthol	2.79 ± 0.26 μM	0.69 ± 0.03	2
	Nicotine	L9′A	0.94 ± 0.07 μM	0.80 ± 0.03	2
	MLA	—	127 ± 22 nM	0.95 ± 0.11	2

Alanine insertion‡	Nicotine	—	0.96 ± 0.07 μM	1.11 ± 0.05	2
	Nicotine	1-mM menthol	1.84 ± 0.18 μM	0.79 ± 0.04	2
	Nicotine	L9′A	0.76 ± 0.09 μM	0.73 ± 0.04	2
	MLA	—	108 ± 12 nM	1.05 ± 0.09	2
Double-glycine insertion‡	Nicotine	—	0.61 ± 0.07 μM	0.75 ± 0.03	3
	Nicotine	1-mM menthol	0.75 ± 0.08 μM	0.76 ± 0.04	2
	Nicotine	L9′A	0.67 ± 0.07 μM	0.77 ± 0.04	2
	MLA	—	187 ± 19 nM	1.03 ± 0.08	4

Hill-equation parameter values. Binding-competition reactions were incubated at 37°C for 24 or 48 h. All individual curves for a given combination of construct and orthosteric-ligand/TMD-perturbation were globally fitted (*24*). The half-competition concentration, the Hill coefficient, and their corresponding errors were estimated from these global fits.

n is the number of individual binding-competition curves generated for each combination of construct and orthosteric-ligand/TMD-perturbation.

* The M2–M3 linker (Fig. 1) of β-GluCl's TMD was replaced by that of the α7-AChR.

† Phe-157 immediately precedes the universally conserved proline (*25*) of the Cys-loop.

‡ The insertion was introduced (in all five subunits) at the C-terminus of the R$^{\underline{227}}$RR pre-M1 linker, between positions 229 and 230.

Table S2.

Background construct	Mutant construct	Expression relative to background (mean ± SE)	*n*
Chicken–C. *elegans* α7-AChR–β-GluCl ECD–TMD chimera	M2–M3 linker replacement*	1.5 ± 0.10†	4
	F157A‡	0.35 ± 0.11†	12
	Arginine insertion (RRRR)	0.66 ± 0.01†	4
	Glycine insertion (**G**RRR)§	0.02 ± 0.004	4
	Double-glycine insertion (**GG**RRR)§	Undetectable	4
	Alanine insertion (RRR**A**)||	0.13 ± 0.003	3
	Double-glycine insertion (RRR**GG**)||	0.10 ± 0.01	4
	Arginine deletion (RR)¶	0.01 ± 0.005	7
	Deletion of Thr-225	Undetectable	6
	Deletion of Met-226	Undetectable	6
Human α7-AChR wild type	Arginine insertion (RRRR)	0.08 ± 0.01	6
	Alanine insertion (RRR**A**)||	0.64 ± 0.10	3
	Double-glycine insertion (RRR**GG**)||	0.36 ± 0.09	5

Plasma-membrane expression level of mutant constructs. Mutant-to-wild-type expression-level ratios higher than 0.01 turned out to be high enough for robust currents to be recorded from fully functional mutants in at least some of the patched cells. Only expression-level ratios lower than 0.01 were considered to be too low. All of the constructs deemed here to be electrically silent expressed above this threshold. For more details, see Materials and Methods.

n denotes the number of replicates.

* The M2–M3 linker (Fig. 1) of β-GluCl's TMD was replaced by that of the α7-AChR.

† From ref. *23*.

‡ Phe-157 immediately precedes the universally conserved proline (*25*) of the Cys-loop.

§ The insertion was introduced (in all five subunits) at the N-terminus of the R$^{\underline{227}}$RR pre-M1 linker, between positions 226 and 227.

|| The insertion was introduced (in all five subunits) at the C-terminus of the R$^{\underline{227}}$RR pre-M1 linker, between positions 229 and 230.

¶ One of the three arginines of the pre-M1 linker was deleted.
